# An Integrated Multimodal-Based CAD System for Breast Cancer Diagnosis

**DOI:** 10.3390/cancers16223740

**Published:** 2024-11-05

**Authors:** Amal Sunba, Maha AlShammari, Afnan Almuhanna, Omer S. Alkhnbashi

**Affiliations:** 1 Information and Computer Science Department, King Fahd University of Petroleum and Minerals, Dhahran 31261, Saudi Arabia; aasunba@pnu.edu.sa (A.S.); mmashammari@iau.edu.sa (M.A.); 2 Department of Computer Sciences, College of Computer and Information Sciences, Princess Nourah Bint Abdulrahman University, Riyadh 11671, Saudi Arabia; 3 Computational Unit, Department of Environmental Health, Institute for Research and Medical Consultations, Imam Abdulrahman Bin Faisal University, Dammam 31441, Saudi Arabia; 4 Department of Radiology, College of Medicine, Imam Abdulrahman Bin Faisal University, Dammam 31441, Saudi Arabia; amuhanna@iau.edu.sa; 5 Center for Applied and Translational Genomics (CATG), Mohammed Bin Rashid University of Medicine and Health Sciences, Dubai Healthcare City, Dubai P.O. Box 50505, United Arab Emirates; 6 College of Medicine, Mohammed Bin Rashid University of Medicine and Health Sciences, Dubai Healthcare City, Dubai P.O. Box 50505, United Arab Emirates

**Keywords:** mammogram, breast cancer, breast tumor, computer-aided diagnosis systems, conventional neural network, machine learning, multi-layer perceptron, disease diagnoses, classification, deep CNN, breast cancer diagnosis, soft voting

## Abstract

Breast cancer has been one of the main causes of death among women recently, and it has been the focus of attention of many specialists and researchers in the health field. Because of its seriousness and spread speed, breast cancer-resisting methods, early diagnosis, diagnosis, and treatment have been the points of research discussion. Many computers-aided diagnosis (CAD) systems have been proposed to reduce the load on physicians and increase the accuracy of breast tumor diagnosis. To the best of our knowledge, combining patient information, including medical history, breast density, age, and other factors, with mammogram features from both breasts in craniocaudal (CC) and mediolateral oblique (MLO) views has not been previously investigated for breast tumor classification. In this paper, we investigated the effectiveness of using those inputs by comparing two combination approaches. The soft voting approach, produced from statistical information-based models (decision tree, random forest, K-nearest neighbor, Gaussian naive Bayes, gradient boosting, and MLP) and an image-based model (CNN), achieved 90% accuracy. Additionally, concatenating statistical and image-based features in a deep learning model achieved 93% accuracy. We found that it produced promising results that would enhance the CAD systems. As a result, this study finds that using both sides of mammograms outperformed the result of using only the infected side. In addition, integrating the mammogram features with statistical information enhanced the accuracy of the tumor classification. Our findings, based on a novel dataset, incorporate both patient information and four-view mammogram images, covering multiple classes: normal, benign, and malignant.

## Simple Summary

Diagnosis of breast cancer goes through multiple processes. Recently, a variety of system-aided diagnosis (CAD) systems have been proposed as primary systems for initial diagnosis based on mammogram screenings. This paper enhances the diagnosis accuracy by using mammograms of both sides of the patient’s breasts instead of the infected side only. In addition, the paper boosts CAD accuracy by adding patient information and medical history along with mammogram images’ features. The proposed multimodal approach will serve as the nucleus for future work at both data and system levels to diagnose breast cancer and other diseases caused by various factors.

## 1. Introduction

**Figure 1:**
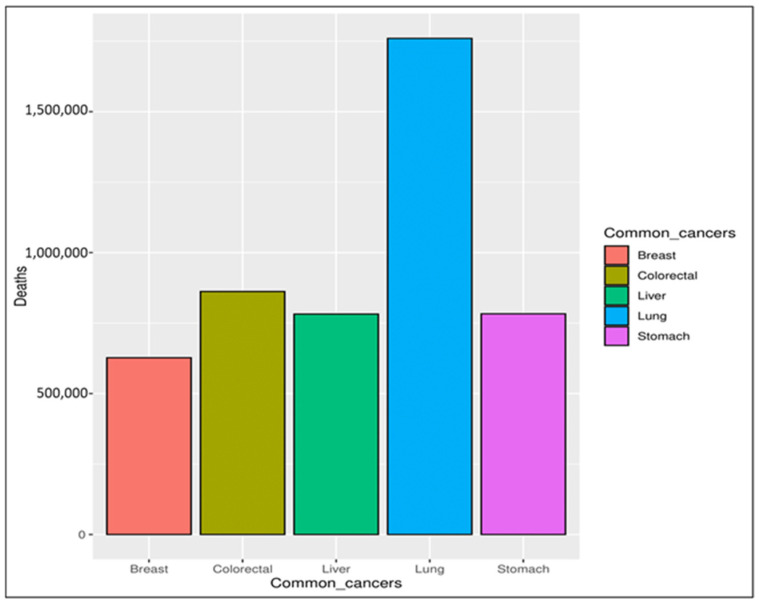
Common causes of cancer deaths.

In recent decades, cancer has been considered a significant threat to human life, with an expectation that it will become one of the leading causes of death in the following decades [1]. Cancer is a group of more than one hundred diseases that may affect any part of the body, such as skin, blood, or other body tissues [2,3]. A tumor that is malignant grows and spreads throughout the body [3,4], whereas one that is benign stays the same size. Any cancer can spread throughout the body to other areas via either the lymphatic or venous system [5]. Obesity, excessive alcohol intake, and tobacco use are the main risk factors for cancer infections. Depending on the symptoms, it can be detected via a multi-stage process incorporating clinical examination, imaging, laboratory tests, etc. Regardless of the examination, the final diagnosis is made based on a pathologist’s opinion [4,6]. According to the World Health Organization (WHO) [2], the mortality rate caused by different types of cancer was 9.6 million in 2018, with breast cancer ranked as the fifth most common cause of cancer deaths, as seen in Figure 1. Breast cancer is rated the second most common cause of women’s death [7,8,9]. It affects both women and men, but it is more common in women [10,11]. The main symptoms indicating potential breast cancer include a change in the skin of the breast to look similar to the skin of an orange, thickness in the breast tissue, pain in the armpits, and a change in the shape and size of the breast [12,13].

A practical and reliable tool for breast imaging is the mammogram. Although it has some limitations related to radiation dose and image quality, it is frequently used for breast cancer detection [14,15]. These limitations have been improved by replacing the X-ray film (the conventional mammogram) with a solid-state detector. It converts the image into electrical signals. In the new electrical signals form, the imaging process can reduce the radiation dose and improve the image quality. These kinds of detectors are similar to those in a digital camera. The generated electrical signals can be seen on the screen or printed as a conventional mammography film [14,15]. Mammograms typically utilize two key views: the mediolateral oblique (MLO) view and the craniocaudal (CC) view. The MLO view captures breast tissue from the top down at an angle, providing a comprehensive view of the breast, including the axillary region, which is crucial for detecting tumors that may not be visible in other views. The CC view, on the other hand, captures the breast from a straight-on perspective, focusing on the inner breast tissue. Together, these views enhance the accuracy of breast cancer detection, allowing radiologists to analyze the full anatomy of the breast more effectively.

Due to the increased detection of breast cancer in its late stages, which affects the mortality rate, there is a worldwide attempt to raise awareness of the benefits of early detection of breast cancer. Mammography images produce a tremendous amount of information that a radiologist has to analyze and assess comprehensively in a short time in order to detect the type of tumor and what stage it is at. At the same time, the necessary multistage process usually takes a long time. Thus, many researchers in this area are proposing computer-aided diagnosis (CAD) systems to detect or classify such tumors with highly accurate results in a reasonable time [16]. Such systems can save the patient from going through the multistage process to detect the tumor and make the treatment decision faster, contributing to saving the patient’s life. This presents researchers and physicians with a significant challenge to propose a system that can meet the above requirements. Accordingly, this paper proposes a mammography-based deep learning technique that uses four views per case for multi-class diagnoses: normal, benign, and malignant.

For more elaboration, we describe the three classes. Normal tissue refers to healthy, non-cancerous breast tissue. When compared to tumors, normal tissues show no signs of abnormal cell growth or irregularities. Benign tumors are non-cancerous and do not spread to other parts of the body. They typically have well-defined borders and tend to grow slower than malignant tumors. Benign tumors are usually not life-threatening. On the other hand, malignant tumors are cancerous and have the potential to spread to other tissues and organs, a process known as metastasis. They tend to grow quickly and have irregular borders. Malignant tumors can be life-threatening if not treated promptly. Therefore, characteristics such as shape, size, and borders of the tumor, in addition to the tissue structure of the breast, are evaluated to distinguish between benign, malignant, and normal tissues [17].

As the CAD system for computer-aided diagnosis of breast cancer has been of interest to many researchers in the field, they have proposed various methodologies, such as solutions relying on mammogram preprocessing or deep learning algorithms and other techniques [17]. In addition, many studies have introduced novel solutions that integrate multiple techniques into a single phase, potentially leading to positive outcomes [18].

Prior research employed machine learning with binary classification to identify certain cancers, including lungs, brain, stomach, skin, kidney, and breast cancers [19].

Our work addresses a specific gap identified in our review: the lack of a combined approach that integrates patient-specific statistical information, such as age, tissue density, biopsy history, and health status, with four-view mammogram images (CC and MLO) for each breast. This integration offers critical insights into a patient’s history that can directly impact cancer risk, while the four-view mammograms provide a comprehensive perspective on breast tissue structure and potential asymmetries. Early predictions based on a single mammogram view is often challenging, particularly in patients with dense breast tissue, where reliable assessment usually requires a team of specialists and further testing. In this paper, we investigated the effectiveness of integrating these data types by comparing two combination approaches. The first approach applied the soft voting approach, produced by statistical information-based models (decision tree, random forest, K-nearest neighbor, Gaussian naive Bayes, Gradient Boosting, and MPL) combined with image-based model using CNN. Meanwhile, the second approach is concatenating these inputs within a deep learning model. Our CAD system aims to improve early diagnostic accuracy and support the identification of high-risk patients who may benefit from more frequent monitoring based on their medical history. This approach aligns with medical practices, potentially streamlining clinical workflows and enhancing patient care through earlier detection and proactive monitoring of at-risk individuals.

Accordingly, this study was designed to answer the following questions based on its formulated hypothesis to address the literature gaps:

RQ#1: How well does CAD perform with the multi-class classification scheme for breast cancer?

Hypos#1: By applying CAD for breast cancer on three classes of mammogram datasets (normal, benign, and malignant), the accuracy of detecting breast lesions (abnormal-ity) can be enhanced.

RQ#2: How does the number of mammography views of each case affect the accuracy of the diagnosis?

Hypos#2: By utilizing CC and MLO mammography views for both breasts for the case, the precision of the diagnosis system is increased.

RQ#3: How does the text on the mammograms affect the results and correctness of the diagnoses?

Hypos#3: By training CNN with mammograms containing text, the model fails to detect breast lesions correctly.

RQ#4: How does the statistics information dataset affect the accuracy of the CAD for breast cancer?

Hypos#4: By combining the statistics information dataset features and mammogram features, the accuracy of the CAD is enhanced.

## 2. Literature Review

Many researchers proposed mammography-based CAD systems for breast cancer diagnosis. In this section, we review recent works relevant to our study, identifying gaps in the existing body of knowledge and constructively assessing the methodologies and outcomes. Our review process considered six key factors: mammography views, dataset size, type of classification, image preprocessing techniques, feature extraction algorithms, and classification models. Table 1 summarizes the techniques and limitations of previous work and highlights how our proposal advances these methodologies.

Albalawi et al. applied conventional image preprocessing and classification techniques in their proposed CNN classifier, utilizing the MIAS dataset and mammography images with MLO views [20]. Wiener filtering and k-means clustering segmentation were applied before feature extraction and classification, comparing the model’s performance to conventional techniques such as ANN, DNN, SVM, and LSVM. While their model outperforms these alternatives with a slight improvement of 0.7–3.4% in accuracy, larger datasets are needed to generalize the findings. Similarly, Escorcia-Gutierrez et al. proposed an ADL-BCD for breast lesion detection using mammograms, incorporating ResNet34 and wavelet neural networks [21]. Their study, limited to the MIAS dataset, also highlights the need for larger datasets for comprehensive validation.

Muduli et al. took a different approach by proposing a CNN model with fewer learnable layers, thus reducing computational complexity [22]. Although validated across five datasets, including three mammograms and two ultrasounds, the model’s success suggests that further exploration of diverse image processing techniques could enhance results. In a related effort, Gargour et al. developed a model based on optimized texture and shape features, leveraging both ML and DL techniques for tumor mass classification, termed C(M-ZMs) [23]. Their method involves segmenting the tumor region using RG and morphological operators before extracting shape features with Zernike moments and texture features with a monogenic-local binary pattern. Validation with 520 mammograms from a DDSM dataset showed that their CNN classifier achieved 99.5% accuracy, indicating that fusion features significantly outperformed individual shape or texture features, especially in challenging cases with lesions in dense breast tissues. Similarly, Kavitha et al. focused on image preprocessing techniques, such as fuzzy-based median filtering and tumor region segmentation using various algorithms, to improve breast cancer classification accuracy [24,25].

Furthermore, Punitha et al. introduced a hybrid optimization algorithm for feature selection and ANN parameter tuning, which improved breast cancer diagnosis performance, achieving up to 99.2% accuracy across multiple datasets [26]. However, their results demonstrate the trade-off between accuracy and diagnosis complexity, highlighting the challenge of balancing computational efficiency with classification performance.

A recent study by Ahmad et al. presented a significant advancement in breast cancer diagnosis through the development of a deep learning-powered CAD system [27]. The CAD system enhances the detection and classification of breast abnormalities by utilizing a combination of advanced DL models, including YOLO for detection, Associated-ResUNets for segmentation, and a customized AlexNet-based model for classification (BreastNet-SVM). This integrated framework achieves an impressive overall classification accuracy of 99.16% and demonstrates a 99% success rate in identifying and categorizing breast lesions.

In this context, the paper by Dada et al. proposes a CAD system for breast cancer detection from mammogram images, focusing on the application of DL techniques [28]. The study investigates the prediction accuracy of three distinct models (CNN, Inception, and EfficientNet) on a commonly used breast cancer dataset, specifically classifying images into benign, malignant, and normal categories. The primary objective was to evaluate the accuracy of these networks using the same dataset and assess the consistency of their predictions. The findings reveal that EfficientNet outperformed the other models, achieving an impressive overall accuracy of 98.29%.

Dina A. Ragab et al. conducted four experiments using end-to-end fine-tuned DCNNs for feature extraction combined with SVM for mammographic classification. Their approach involves feature fusion from multiple networks to improve classification accuracy. Additionally, they employed PCA to reduce the feature space and computational cost without significantly affecting accuracy. The highest accuracy achieved was 97.9% for the CBIS-DDSM dataset and 97.4% for the MIAS dataset using fused deep features and SVM classifiers. PCA significantly reduced execution time, making the classification process more efficient [29].

Additionally, Dina A. Ragab et al. developed a CAD system for mammogram images, enhancing them using CLAHE to improve visibility. The methodology includes removing the pectoral muscle and suppressing artifacts to isolate the ROI. They extracted statistical features (entropy, mean, variance, standard deviation, range, minimum, maximum, and root mean square) to form a feature vector for classification. Two feature selection techniques, best first and random search, were used to improve model accuracy. The study employed individual classifiers (k-NN, DT, RF, and RT) and multiple classifier systems utilizing ensemble techniques such as bagging and AdaBoosting. Augmentation methods, such as rotation and flipping, were applied to address class imbalance, enhancing the representation of the abnormal class [30].

Maqsood et al. proposed transferable texture CNNs, which utilize multiple DL models for feature extraction [31]. Jayandhi et al. also demonstrated that combining DL architectures (such as VGG) with SVM could efficiently classify mammogram images [32]. These approaches show that combining different techniques can enhance breast cancer diagnosis models. Finally, Sun et al. designed a multi-view network that extracts features from CC and MLO views, demonstrating the advantage of multi-view feature extraction on classification accuracy using the DDSM dataset t [33]. This underscores the importance of leveraging multiple views for improved diagnostic performance.

While these studies primarily focus on image preprocessing, feature extraction, and classification techniques, they lack the integration of patient-specific statistical data such as age, breast density, biopsy history, and medical status. None of the reviewed studies addressed combining statistical information with both mammogram views (CC and MLO) for both breasts of each patient, nor did they investigate the effects of radiologist text annotations on the classification performance of CAD systems, despite the fact that this text is consistently present on mammography images. Moreover, most of these models focus on binary classification (malignant vs. benign), leaving a gap in research on multi-class classification systems that include normal cases.

Lulu Wang’s recent review on CAD techniques for breast cancer outlines various preprocessing methods and models used in mammography [34]. However, it significantly overlooks critical gaps in the research. The review does not address the integration of patient-specific statistical data, which could enhance the CAD system’s relevance and accuracy. It also fails to mention the necessity of utilizing both CC and MLO views from both breasts for a more comprehensive assessment. Additionally, the impact of noise and artifacts in mammograms on classification performance is not discussed. These omissions highlight substantial gaps in the current knowledge base, emphasizing the unique contribution of your work, which aims to fill these gaps by incorporating patient data and examining the effects of imaging views and noise on CAD performance.

Our study fills this gap by integrating patient statistics with four-view mammogram images to build a multi-class CAD system. This novel approach not only considers the patient’s history, but also compares the accuracy improvement gained by utilizing both breast sides and addressing the impact of text annotations in mammograms. Thus, our approach extends current research by incorporating patient-specific data and addressing multi-class classification, aiming for a more comprehensive and clinically relevant CAD system.

**Table 1:** Comparative study of CAD for breast cancer.

Ref.	Year	Image Processing	Classifier	Classification	Dataset	Dataset-Type	Dataset Size	Views	ACC	Limitations
[20]	2020	- Wiener filter - K-means clustering	CNN	Binary (benign, malignant)	MIAS	Mammographic images	322 images	-	97.14%	Less accessibility of huge data
[25]	2020	- EM segmentation - WBCT features extraction - SVM–RFE with CBR for features reduction	Q-classifier	Binary (benign, malignant)	- DDSM - MIAS	Mammographic images	1000 images	-	98.16%	-
[26]	2021	- ABC - WOA - RP, LM, and GD - back- propagation	ANN	Binary (benign, malignant)	WBCD, WDBC, WPBC, DDSM, MIAS, INBREAS	Mammographic images	1750 images	-	99.2%	complex and requires more computational time
[23]	2021	- Zernike moments for shape feature extraction - Monogenic-Local Binary Pattern for texture feature extraction Optimized Fusion	CNN SVM ANN KNN	Binary (benign, malignant)	DDSM	Mammographic images	520 images	-	99.5%	Low size dataset
[32]	2021	- convolution filters - Data augmentation	SVM VGG	Binary (benign, malignant)	MIAS	Mammographic images	322 images	-	98.67%	Low size dataset
[24]	2021	- OKMT-SGO for segmentation - CapsNet for feature extraction	BPNN	Binary (benign, malignant)	MIAS, DDSm	Mammographic images	322, 13128	-	98.16%	-
[22]	2021		CNN	Binary (benign, malignant)	- Mammograms (MIAS, DDSM, INbreast) - Ultrasound (BUS-1, BUS-2)	Mammograms ultrasound	MIAS: 50, DDSM: 450, INbreast: 35 BUS-1: 100, BUS-2: 210	-	96.55%,	-
[33]	2022	- cross-entropy for feature extraction - SCL for reduction - Data augmentation	CNN	Binary (benign, malignant)	DDSM	Mammographic images	1371 images	MLO CC	73.55%	lack of publicly available breast mammography databases the insufficient feature extraction ability from breast mammography
[31]	2022	- CLAHE energy layer for texture feature extraction - ECfA for feature selection - CSID fusion algorithm	TTCNN	Binary (benign, malignant)	DDSM, INbreast, MIAS	Mammographic images	DDSM: 981 images INbreast: 269 images MIAS: 119 images	-	99.08%	-
[21]	2022	- Gaussian filter - Tsallis entropy for segmentation - ResNet 34 for feature extraction - COA for Parampara tuning	WNN	multi (normal-benign- malignant)	MIAS	Mammographic images	322 images	-	96.07%	Low size dataset
[29]	2021	- PCA - feature fusion	SVM	Binary (malignant, benign)	CBIS-DDSM, MIAS	Mammograms images	891, 322	-	97.8%	-
[30]	2019	CLAHE, SRG, statistical feature extraction	K-NN, DT, RF, ensemble	Binary classification (normal, abnormal)	MIAS, Digital Mammography Dream Challenge	Mammograms images	322, (34, 466)	-	99.5%	-
[28]	2024	noise reduction, image normalization, and contrast enhancement	CNN, Inception, and EfficientNet.	multi-class (being benign, malignant, and normal)	MaMaTT2	mammogram images.	408	-	98.46%	data accessibility, and the challenge of data imbalance.
[27]	2024	YOLO for Segmentation	BreastNet-SVM	multi-class (being benign, malignant, and normal)	CBIS-DDSM	mammogram images	6165	-	99.16%	the high number of trainable parameters and the need for further validation with larger datasets

## 3. Materials and Methods

This section outlines the dataset preparation and preprocessing steps, followed by a description of our proposed methods for integrating multiple data types using voting and concatenation approaches. Finally, we detail our experimental setup, which includes five traditional ML algorithms and an MLP for classifying statistical data, along with three configurations for the CNN models (unclean images, clean images, and one-breast-side images). Additionally, we present the models and techniques used for combining multi-type data.

### 3.1. Dataset and Preprocessing

In this paper, a raw dataset from King Fahad University Hospital in Khobar, Saudi Arabia was collected. Before this, ethical approval was obtained from the Standing Committee for Research Ethics on Living Creatures at Imam Abdulrahman Bin Faisal University with reference number IRB-2020-13-371 to utilize in this study. The dataset contains 256 records with two parts: statistic information dataset and images dataset. The records were collected according to the cases availability, so no specific criteria as this will restrict the number of records due to the difficulty of accessing medical data. The collected data so far are used to examine our proposed model in the next section. However, collecting more data is in progress to extend our investigation in the future.

#### 3.1.1. Statistic Information Dataset

The collected statistics data includes regular patients’ information, which includes personal information, medical procedures, and tests and mammogram results. Table 2 below identifies the 14 features with their description and selection in our study. Some features were excluded, as there are three features and one is meaningless, which is the patient record ID, and another has the same value for all records, which is nationality. BI-RADS, established by specialists following accepted medical guidelines, is utilized as our classification category. This classification is determined by specialists based on the evaluation of mammogram images and medical records. Consequently, our experiments focus on 11 pertinent features. As the classes are unbalanced, the data augmentation for statistical information was conducted and will be explained in the next section alongside images data augmentation.

#### 3.1.2. Images Dataset

**Figure 2:**
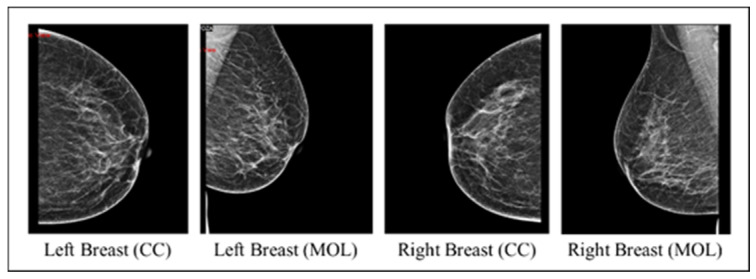
An instance of the four mammography views for a record.

**Figure 3:**
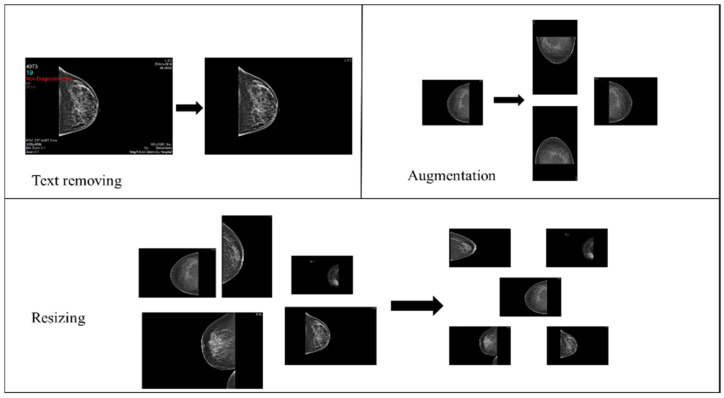
Mammograms preprocessing steps.

The collected images dataset includes four mammography views for each record, with CC and MLO views for both breasts (an instance on Figure 2). The images dataset went through many preprocessing steps as follows:

1. Checking that each record includes MLO and CC views for both breasts.
2. Image cleaning: patient information and radiologist comments on mammograms were deleted manually using paint tools and Photoshop to preserve image quality.
3. Image resizing: The original mammograms’ dimensions vary due to sources and devices. Therefore, Photoshop was used to resize all mammograms into 1800 × 1800.
4. Data augmentation: The dataset initially consisted of 100 records from BI-RADS-1, 100 from BI-RADS-2, and only 56 from BI-RADS-5, creating an imbalance among these classes. To address this, random rotation and flipping were applied to BI-RADS-5 mammograms for data augmentation, increasing its records to 100. For the statistical data, slight random alterations were introduced to preserve feature integrity while balancing the classes. Figure 3 provides an overview of the preprocessing steps for the images.

### 3.2. Proposed Method

This study proposed a novel technique to enhance the performance of the CAD system for breast cancer diagnosis. According to S. Łukasiewicz’s review, many factors increase the risk of breast cancer, including age, genetics and racial factors, breast density, family history, and previous health history [35]. Therefore, this work takes advantage of a statistical information dataset and combines its features with the image features from mammogram datasets.

The study proposed two multi-class classification methods that integrate statistics and image features. In the first method, a convolutional neural network (CNN) is trained using features from the mammograms while utilizing traditional ML algorithms for analyzing and categorizing statistical information. Then, a soft voting technique is applied to the CNN classifier output and the outperforming ML classifier output for the final output.

The soft voting is one of the ensemble strategies used to create a potent classifier with greater classification accuracy than conventional ML classifiers. On the majority of datasets, ensemble-based techniques frequently outperform alternative algorithms. This approach uses many artificial intelligence models and votes among their prediction results to construct a powerful model that carries the strength of the input models [36]. Due to different types of data being integrated in this study, images and statistical information, this approach would be advantageous.

**Figure 4:**
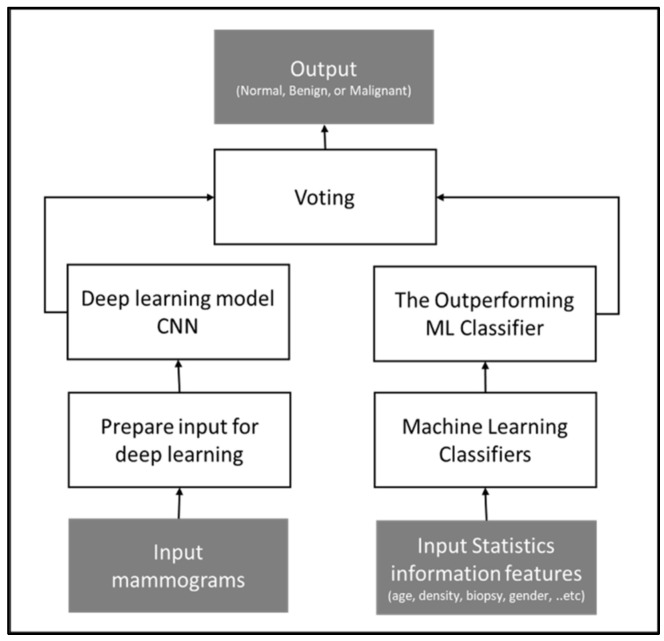
Conceptual framework of voting approach, outputting the tumor diagnosis class based on the voting technique applied to two models: statistical-based and image-based.

Figure 4 illustrates the conceptual framework of the method.

**Figure 5:**
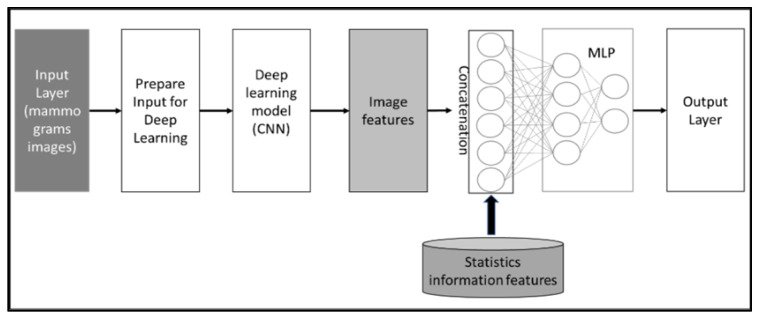
Conceptual framework of features concatenation approach, which extracts features from mammogram images and then concatenates these extracted features with statistical features.

The second method utilizes CNN as well for extracting image features from the mammogram datasets and concatenates the extracted features from images with statistical information features to predict case classes. Figure 5 illustrates the conceptual framework of the method. Both approaches enhance the diagnosis performance of the system.

#### 3.2.1. Experiment Setup

For our study, the dataset was divided as follows: 90% for training and validation and 10% for testing using 5-fold cross-validation (CV). It is important to note that the test data were separated before applying data augmentation techniques. Specifically, this resulted in 30 samples designated for testing, while 270 samples were allocated for training and validation. Within each fold, 20% of the 270 samples (54 samples) were used for validation, leaving 216 samples for training. Moreover, we utilized default settings for the classifiers, so we are not discussing the specifics of these settings further in our methodology.

Regarding performance evaluation, we assessed model performance using accuracy, F1-score, recall, precision, and AUC-ROC. However, to maintain simplicity and readability, this paper primarily focuses on accuracy comparisons across experiments.

Further details regarding classifiers and comprehensive tables with metrics for each experiment are available in the Supplementary File.

##### Statistical Information-Based Model

Different supervised ML algorithms are applied for classification: decision tree (DT), random forest (RF), K-nearest neighbor (KNN), Gaussian naive Bayes (GNB), and gradient boosting (GB). In addition, A multi-layer perception (MLP) is applied with the following parameters: (loss: “sparse categorical_crossentropy”, optimizer: “Adam”, layers: three). Hence, the outperforming classifiers are applied on the hybrid proposed models.

##### Mammograms-Based Model

CNN is utilized for image classification. It automates feature extraction from images, learns image representation, and it is widely used for image recognition tasks in medical image analysis [37]. In the image processing section, a panda data frame is used to read the data. The images are resized to 512 and cropped. Each case’s images are appended in the image array to get them collected as one case input. Then, VGG19 is utilized with the default setting, except for freezing the first layer, to prevent overfitting during training while maintaining the accuracy. To investigate the effects of the image’s conditions on the breast tumor classification performance, the model is executed through a systematic approach:

1. Uncleaned images: the model processes images containing text, with four mammograms for each patient, covering two views per breast side.
2. Cleaned images: the same model is then applied to images devoid of text, also featuring four mammograms per patient and two views per breast side.
3. The outperforming scenario between the uncleaned and cleaned images is rerun using only two images from the two views of the affected breast side.

Thereafter, the case of mammogram images that achieve the highest accuracy is applied in the hybrid proposed models.

##### Combining Approaches

In this step, the CNN model with the best performing mammogram data settings is combined with the best statistical information classifiers. Different approaches are tested for the integration:

- Soft voting approach: The idea of the model proposed by B. Kurian, which ensembles classifiers by soft voting and hard voting, is applied to the utilized classifiers in this study [38]. Soft voting is applied to combine the predictions of the CNN and the ML models by taking the weighted average of their predicted probabilities. In soft voting, the models are trained and then used to make predictions for the test data. The probabilities of each predicted class are averaged across the models to produce the final tumor class prediction. This can improve the accuracy and robustness of the ensembled models, especially when individual models perform well on different data subsets.
- Hard voting approach: Hard voting is a simple ensemble technique where multiple models are trained separately on the same data and their individual predictions are combined by majority voting. The final prediction for the tumor class is determined by the most common prediction among the individual models. This approach simplifies the integration process while leveraging the strengths of each model.
- Concatenating approach: The concatenation between CNN and MLP refers to the combination of the two neural network architectures. This can be achieved by taking the output of the CNN and feeding it into a fully connected layer (from the MLP). Then, the MLP can learn from features extracted by the CNN in conjunction with statistical data features, potentially improving the overall performance of the model. This approach is commonly used in deep learning applications, such as image recognition. For example, D. Kwon successfully combined a CNN model using panoramic views with an MLP model utilizing patient clinical data to predict the timing for extracting a mandibular third molar tooth, achieving impressive accuracy in clinical practice [39].

For the experimental setup of the experiments, the following settings were on the computer that was running Google Collab:

- Processor: Intel(R) Core (TM) i7- 10510U CPU 1.80 GHz
- RAM: 16.00 GB
- OS Edition: Windows 11 Home

## 4. Results and Discussion

**Figure 6:**
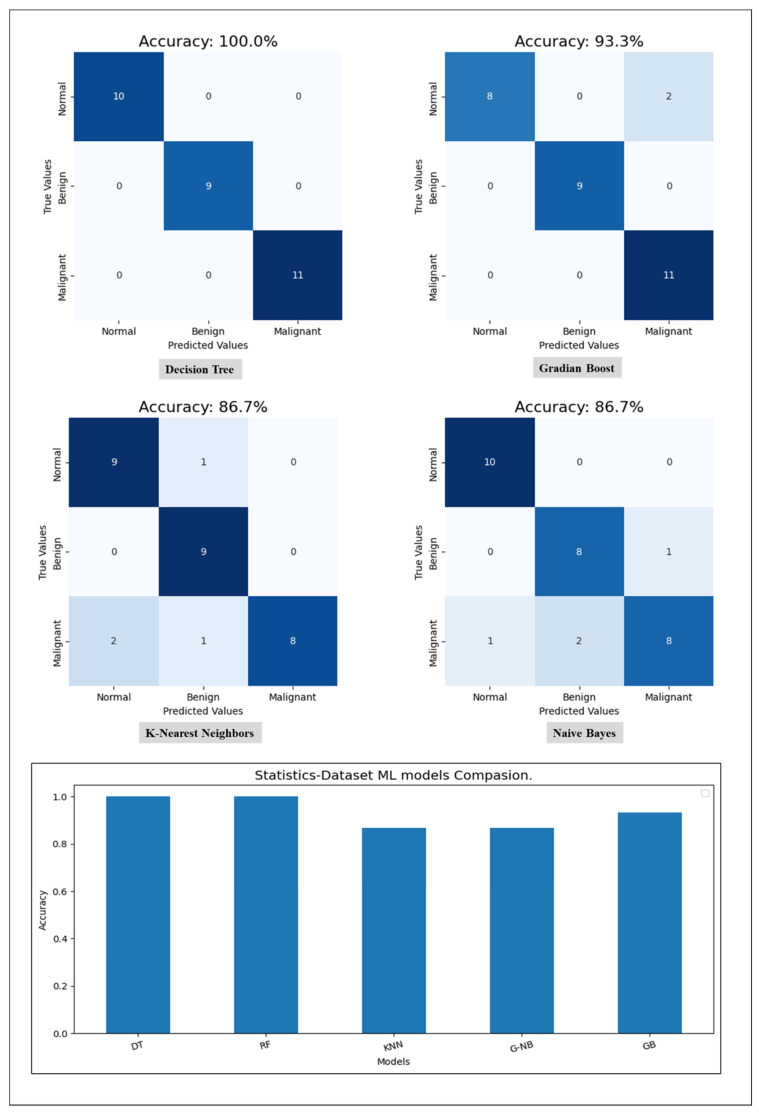
A comparison of ML classifiers (DT, RF, KNN, GNB, and GB) on statistical information resulting from the test set.

**Figure 7:**
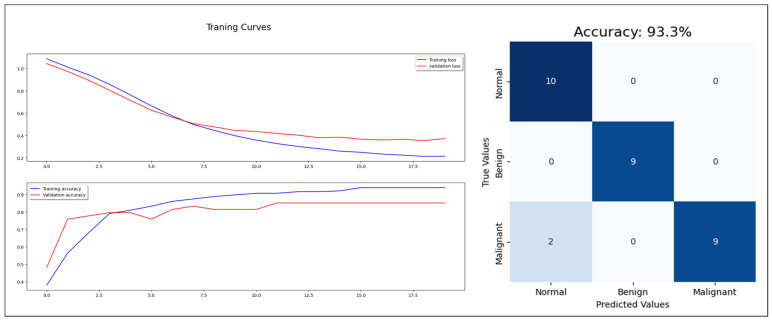
MLP model training, validation, and confusion matrix resulting from test dataset.

Starting with the statistical information dataset, the training went smoothly with the applied ML classifier in addition to the MLP model. Furthermore, the models showed different outperforming on each tumor class reorganization. DT and RF classified the entire test sample correctly. KNN performed well on the benign tumors while failing on 30% of the malignant tumor. Figure 6 shows a comparison of the performance of the ML classifiers. Similarly, the proposed MLP shows good performance, especially with normal and benign cases recognition with 98% AUC-ROC and 93.3% accuracy, which is shown on Figure 7. Hence, the statistical information produced good results, which can be used to boost tumor classification in mammography.

**Figure 8:**
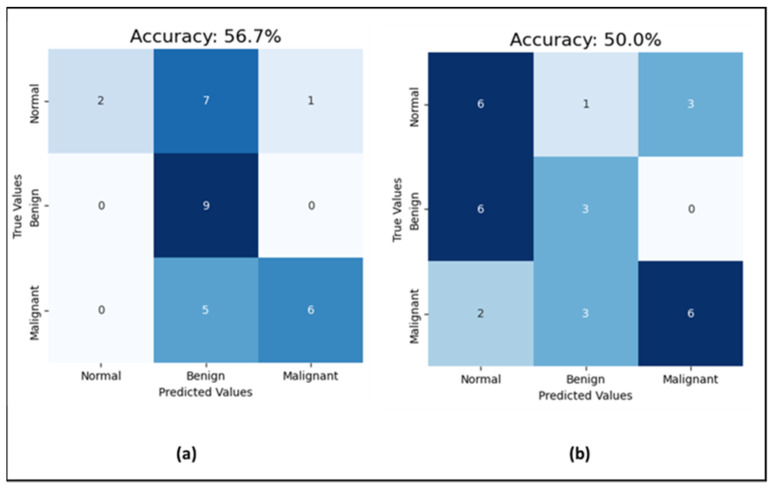
Results of the test set (**a**) the CNN performance with cleaned mammograms; (**b**) the CNN performance with uncleaned mammograms.

Moving to the CNN model for mammogram classification, the experiment went through three phases. First, for investigating the effect of noise in the mammograms on the classification task, the model was run with uncleaned images that contained dates, notes, and patient information, and then with cleaned images without text. The prediction accuracy jumped from 50% with unclean images to 56.7% with clean images (which is shown in Figure 8), while the AUC-ROC remained consistent at 71% for both. Additionally, the F1-score improved from 50% to 56% with clean images. This indicates that data cleaning positively impacts both the model’s prediction accuracy and F1-score, enhancing its precision and recall, while the stable AUC-ROC suggests a consistent ability to distinguish between classes across conditions. As emphasized by S. Ghafari, the CNN model may perform better with clean mammograms because the presence of text could introduce noise or irrelevant information that may confuse the model [40]. The model might be trained on images of mammograms alone, without text labels or annotations, and it would thus be better adapted to recognize and classify abnormalities or features based on the mammogram image only. Adding text information may also change the distribution of the data, making it harder for the model to generalize and perform well on new examples. Therefore, the next experiments were completed with clean mammogram datasets.

**Figure 9:**
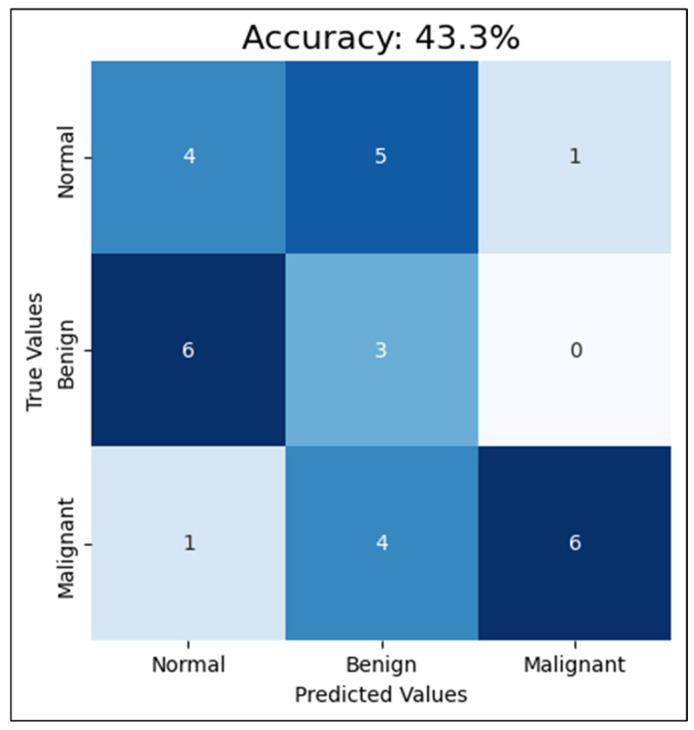
The CNN performance using the test set with one-side breast.

Second, compared with training the CNN with four mammograms, MLO and CC for both breast sides and only one breast side, using the mammograms of one breast side only reduced the accuracy to 43%, F1-score to 45.7%, and AUC-ROC to 68.1%. This reduction in the performance clarifies how excluding the other side limits the model’s exposure to diverse tissue patterns, diminishing its diagnostic effectiveness. Detailed classification results among the classes are shown in Figure 9. This proves that training a CNN model with mammograms of both breasts provides more information and features for the model to learn and detect patterns related to breast cancer. According to the R. Gerami review, “no single imaging modality can detect and characterize the majority of breast lesions” [41]. Therefore, using both breasts in the training data, the model can learn to distinguish normal and abnormal patterns that may only appear in one breast, or may be present in both, but with varying degrees of severity. Additionally, since mammograms of both breasts are typically taken during routine screening, using images from both sides for training can more accurately reflect real-world scenarios and enhance the model’s ability to detect breast cancer. This approach aligns with clinical practices, as physicians often analyze bilateral mammograms to identify asymmetries and potential abnormalities, thereby improving diagnostic accuracy. Additionally, training the CNN model with only one breast side limits the amount of information and patterns that the model can learn from. This can lead to lower accuracy rates for cancer detection in mammograms. Based on these findings, CNN trained with cleaned mammograms for two breast sides (four images) was utilized in the next experiment, which is the models’ integration phase.

In the soft-voting approach, we observed that performance varied based on the weights assigned to each model, with CNN processing image data and other classifiers handling statistical features. To ensure a fair contribution between image and statistical information, we maintained an equal distribution of weights: 0.5 for the CNN and 0.5 spread across outperformed statistical classifiers. An initial configuration with weights (CNN: 0.5, DT: 0.1, KNN: 0.1, NB: 0.1, GB: 0.1, and MLP: 0.1), achieved an accuracy of 40% and an AUC-ROC of 97%. By adjusting weights (CNN: 0.4, MLP: 0.13, GB: 0.15, NB: 0.05, KNN: 0.1, and DT: 0.15), the model achieved its highest performance with 90% accuracy and 100% AUC-ROC. This gradual tuning highlights the impact of balancing image and statistical features on model performance. Additional configuration details and the rationale for these adjustments are provided in the Supplementary File. On the other hand, the hard voting output was derived by taking the most common prediction among MLP, NB, and CNN, resulting in 93% accuracy. Since we have only one model trained on images and six models trained on statistical features, we mitigated potential bias by including the lower-performing statistical model, NB, alongside MLP and CNN in the hard voting ensemble.

**Figure 10:**
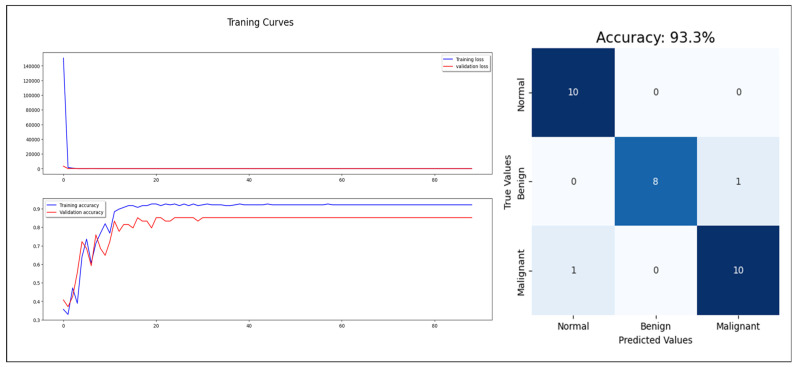
The concatenated model training, validation, and test.

Regarding the concatenating approach, the model enhanced the classification performance with 93% accuracy, 93% F1-score, and 98% AUC-ROC (see Figure 10). The three approaches proved the benefits of combining the extracted features from mammogram images with the statistical features, which reflect the factors used in the breast cancer diagnosis in the real world. Combining mammograms with patient information in the CAD for breast cancer can improve the accuracy of tumor classification because it allows the classifier to consider a wider range of factors that may affect the likelihood of a patient having a particular type of tumor. For example, including information about a patient’s age, family history, and lifestyle habits can help the classifier differentiate between tumors that are likely to be benign or malignant, or to be sensitive or resistant to certain treatments. Additionally, by analyzing both the mammograms and patient information, the classifier can learn patterns and relationships between the data that would be difficult or impossible to detect otherwise. This helps the classifier to make more accurate predictions about the most likely tumor classification, reducing the potential for false positives or false negatives and improving overall diagnostic accuracy. Additionally, by taking a holistic approach to tumor classification, the classifier can better account for individual differences in patient health and biology, leading to more personalized and effective treatment options.

Although our dataset size is rational compared to the state-of-the-art in CAD systems, we recognize it as an internal threat to our study’s validity. To address this limitation, we selected learning techniques that are particularly effective for small datasets. We utilized the VGG19 model, a deep learning neural network pre-trained on a large image dataset, which is especially advantageous for achieving reliable results with limited data. Additionally, we implemented cross-validation to enhance the robustness of our results, ensuring that the model’s performance is consistent and generalizable across different subsets of data. The effectiveness of these strategies was confirmed by the convergence of validation and testing results across all metrics, particularly for the model trained with images and statistically merged features. Furthermore, our experiments demonstrated that each of the three multi-data combinational techniques significantly boosted the overall model performance.

In validating our approach, we combined statistical data with mammogram images, using six distinct experiments and cross-validation to rigorously assess model performance across different data types. Our results show that models trained on clean images outperformed those trained on unclean images, and integrating images with statistical information further improved diagnostic accuracy. We applied a 5-fold cross-validation and computed 95% confidence intervals for all key performance metrics, demonstrating consistency and reliability across tests. Additionally, we used a paired t-test to evaluate the significance of differences between various model configurations. Notably, the comparison between models trained solely on images and those trained on combined features yielded significant improvement (*p*-value = 0.001798), underscoring the effectiveness of our integration approach. These findings affirm that integrating complementary data sources enhances model performance, with promising implications for its applicability in clinical settings. The complete validation details, including confidence intervals and performance metrics, are provided in the Supplementary File.

## 5. Conclusions

This paper proposes a novel approach to enhance the reliability and efficiency of CAD for breast cancer diagnosis. By integrating patient-specific medical, genetic, cultural, and breast density information with mammography features, our method more realistically simulates the real-life scenario of diagnosing breast cancer. The three integrated model approaches showed promising results, with accuracy rates ranging from 90% to 93%, compared to a maximum of 57% accuracy when using images alone. These findings suggest that CAD systems can be made significantly more effective, not only for breast cancer, but potentially for other diseases as well.

In addition, the paper demonstrates the impact of noise from radiologists’ text annotations on mammogram images, as well as the effect of including mammogram views from both left and right breasts, on the system’s accuracy in classifying tumor type. Cleaning the images by removing text annotations led to a notable improvement in accuracy (from 50% to 57%), while using only one breast side reduced accuracy significantly by 13%.

To clarify, this work is primarily focused on the technical perspective, with medical insight provided by a radiologist on our team. In our study, we specifically augmented the malignant class due to its rarity in comparison to the abundant benign and normal cases within the dataset. Our main focus is on integrating statistical patient information with mammogram image features to assess its impact on prediction performance. The initial findings are promising, indicating performance improvements. Notably, our study is unique in its objective, with no direct comparisons available since prior studies did not pursue the same goal. We acknowledge the importance of addressing class imbalance comprehensively and plan to explore potential improvements in future research.

In the future, we plan to integrate more images into the classifiers to improve accuracy. Additionally, we aim to incorporate data from various BI-RADS classes. Our method could also be developed into a real-time CAD system to support diagnostic processes for physicians and radiologists.

## Figures and Tables

**Table 2:** Features selection from the statistic information dataset.

	Feature	Type	Description	Feature Selection	Modifications
**1**	Ser	Numeric	Serial number	Yes	-
**2**	MRN	Numeric	Patient’s record ID	No	-
**3**	Age	Numeric	Patient’s age	Yes	
**4**	Menopause	Numeric	Menopause status. 0 = pre, 1 = post	Yes	-
**5**	Gender	Numeric	Male or female	Yes	-
**6**	Year	Numeric	0 = 2014, 1 = 2015, 2 = 2016, 3 = 2017, 4 = 2018	Yes	-
**7**	CC	Numeric	Patient’s complaint (0 = screening, 1 = pain, 2 = lump, 3 = scar, 4 = moles, 5 = skin retraction, 6 = tissue thickening, 7 = nipple discharge, 8 = nipple inversion or retraction, 9 = other	Yes	-
**8**	Age_menarche	Numeric	Age of menarche	Yes	-
**9**	Age_1st_birth	Numeric	Age of first birth	Yes	-
**10**	HRT	Numeric	taking HRT. 0 = no, 1 = yes	Yes	-
**11**	Fhx	Numeric	Family history. 0 = no, 1 = yes	Yes	-
**12**	Nationality	String	Nationality. s = Saudi	No	-
**13**	Density	String	Breast density. A = A, B = B, C = C, D = D	Yes	Changed to numeric values. A = 1, B = 2, C = 3, D = 4
**14**	Bi_RADS	String	Bi-RADS. 1 = 1, 2 = 2, 5 = 5	Yes	-
**15**	Biopsy	String	Biopsy. 0 = normal, 1 = malignant	Yes	-

## Data Availability

The data are not publicly available due to patients’ privacy. It was obtained from King Fahad University Hospital in Khobar, Saudi Arabia.

## Supplementary Materials

The following supporting information can be downloaded at: https://www.mdpi.com/article/10.3390/cancers16223740/s1.

## References

[1] Tang J., Member S., Rangayyan R.M., Xu J., El Naqa I. (2009). Computer-Aided Detection and Diagnosis of Breast Cancer with Mammography: Recent Advances. IEEE Trans. Inf. Technol. Biomed..

[2] WHO (2022). Cancer. [Online]. https://www.who.int/news-room/fact-sheets/detail/cancer.

[3] Cherath L., Sullivan M. (2014). Cancer. The GALE ENCYCLOPEDIA of Science.

[4] Odle D.D.T.G., Davidson A.M.T. (2008). Cancer. The Gale Encyclopedia of Alternative Medicine.

[5] Karaman S., Detmar M. (2014). Mechanisms of lymphatic metastasis. J. Clin. Investig..

[6] Kaplan W. (2013). Cancer and Cancer Therapeutics. Priority Medicines for Europe and the World.

[7] Chaurasia V., Pal S. (2017). A Novel Approach for Breast Cancer Detection using Data Mining Techniques. Int. J. Innov. Res. Comput. Commun. Eng..

[8] Han S., Kang H.-K., Jeong J.-Y., Park M.-H., Kim W., Bang W.-C., Seong Y.-K. (2017). A Deep Learning Framework for Supporting the Classification of Breast Lesions in Ultrasound Images. Phys. Med. Biol..

[9] Kele A., Kele A., Yavuz U. (2011). Expert system based on neuro-fuzzy rules for diagnosis breast cancer. Expert Syst. Appl..

[10] Ly D., Forman D., Ferlay J., Brinton L.A., Cook M.B. (2013). An international comparison of male and female breast cancer incidence rates. Int. J. Cancer.

[11] Alotaibi R.M., Rezk H.R., Juliana C.I., Guure C. (2018). Breast cancer mortality in Saudi Arabia: Modelling observed and unobserved factors. PLoS ONE.

[12] Shah R. (2014). Pathogenesis, prevention, diagnosis and treatment of breast cancer. World J. Clin. Oncol..

[13] Society A.C. (2018). About Breast Cancer.

[14] Nees A.V. (2008). Digital Mammography. Are There Advantages in Screening for Breast Cancer?. Acad. Radiol..

[15] Feig S.A., Yaffe M.J. (1995). Digital mammography, computer-aided diagnosis, and telemammography. Radiol. Clin. North Am..

[16] Guo Z., Xie J., Wan Y., Zhang M., Qiao L., Yu J., Chen S., Li B., Yao Y. (2022). A Review of the Current State of the Computer-Aided Diagnosis (CAD) Systems for Breast Cancer Diagnosis. Open Life Sci..

[17] Jalalian A., Mashohor S., Mahmud R., Karasfi B., Iqbal M., Saripan B., Rahman A., Ramli B. (2017). Foundation and Methodologies in Computer-Aided Diagnosis Systems for Breast Cancer Detection. Excli. J..

[18] Alshammari M.M., Almuhanna A., Alhiyafi J. (2022). Mammography Image-Based Diagnosis of Breast Cancer Using Machine Learning: A Pilot Study. Sensors.

[19] Arika R.N., Mindila A., Cheruiyo W. (2022). Machine Learning Algorithms for Breast Cancer Diagnosis: Challenges, Prospects and Future Research Directions. J. Oncol. Res..

[20] Albalawi U., Manimurugan S., Varatharajan R. (2020). Classification of breast cancer mammogram images using convolution neural network. Concurr. Comput. Pract. Exp..

[21] Escorcia-Gutierrez J., Mansour R.F., Beleño K., Jiménez-Cabas J., Pérez M., Madera N., Velasquez K. (2022). Automated Deep Learning Empowered Breast Cancer Diagnosis Using Biomedical Mammogram Images. Comput. Mater. Contin..

[22] Muduli D., Dash R., Majhi B. (2021). Automated diagnosis of breast cancer using multi-modal datasets: A deep convolution neural network based approach. Biomed. Signal Process. Control.

[23] Gargouri N., Mokni R., Damak A., Sellami D., Abid R. (2021). Combination of Texture and Shape Features Using Machine and Deep Learning Algorithms for Breast Cancer Diagnosis. Res. Sq..

[24] Kavitha T., Mathai P.P., Karthikeyan C., Ashok M., Kohar R., Avanija J., Neelakandan S. (2021). Deep Learning Based Capsule Neural Network Model for Breast Cancer Diagnosis Using Mammogram Images. Interdiscip. Sci.—Comput. Life Sci..

[25] Eltrass A.S., Salama M.S. (2020). Fully automated scheme for computer-aided detection and breast cancer diagnosis using digitised mammograms. IET Image Process..

[26] Stephan P., Stephan T., Kannan R., Abraham A. (2021). A hybrid artificial bee colony with whale optimization algorithm for improved breast cancer diagnosis. Neural Comput. Appl..

[27] Ahmad J., Akram S., Jaffar A., Ali Z., Bhatti S.M., Ahmad A., Rehman S.U. (2024). Deep Learning Empowered Breast Cancer Diagnosis: Advancements in Detection and Classification. PLoS ONE.

[28] Dada E.G., Oyewola D.O., Misra S. (2024). Computer-aided diagnosis of breast cancer from mammogram images using deep learning algorithms. J. Electr. Syst. Inf. Technol..

[29] Ragab D.A., Attallah O., Sharkas M., Ren J., Marshall S. (2021). A framework for breast cancer classification using Multi-DCNNs. Comput. Biol. Med..

[30] Ragab D.A., Sharkas M., Attallah O. (2019). Breast cancer diagnosis using an efficient CAD system based on multiple classifiers. Diagnostics.

[31] Maqsood S., Damaševičius R., Maskeliūnas R. (2022). TTCNN: A Breast Cancer Detection and Classification towards Computer-Aided Diagnosis Using Digital Mammography in Early Stages. Appl. Sci..

[32] Jayandhi G., Jasmine J.S.L., Joans S.M. (2021). Mammogram Learning System for Breast Cancer Diagnosis Using Deep Learning SVM. Comput. Syst. Sci. Eng..

[33] Sun L., Wen J., Wang J., Zhang Z., Zhao Y., Zhang G., Xu Y. (2022). Breast Mass Classification Based on Supervised Contrastive Learning and Multi-View Consistency Penalty on Mammography. IET Biom..

[34] Wang L. (2024). Mammography with deep learning for breast cancer detection. Front. Oncol..

[35] Łukasiewicz S., Czeczelewski M., Forma A., Baj J., Sitarz R., Stanisławek A. (2021). Breast Cancer—Epidemiology, Risk Factors, Classification, Prognostic Markers, and Current Treatment Strategies—An Updated Review. Cancers.

[36] Hashim M.S., Yassin A.A. (2023). Breast Cancer Prediction Using Soft Voting Classifier Based on Machine Learning Models. Int. J. Comput. Sci..

[37] Puttagunta M., Ravi S. (2023). Medical image analysis based on deep learning approach. Multimed. Tools Appl..

[38] Kurian B., Jyothi V.L. (2023). Breast cancer prediction using ensemble voting classifiers in next-generation sequences. Soft Comput..

[39] Kwon D., Ahn J., Kim C.S., Kang D.O., Paeng J.Y. (2022). A deep learning model based on concatenation approach to predict the time to extract a mandibular third molar tooth. BMC Oral Health.

[40] Ghafari S., Tarnik M.G., Yazdi H.S. (2021). Robustness of convolutional neural network models in hyperspectral noisy datasets with loss functions. Comput. Electr. Eng..

[41] Gerami R., Joni S.S., Akhondi N., Etemadi A., Fosouli M., Eghbal A.F., Souri Z. (2022). A Literature Review on the Imaging Methods for Breast Cancer. Int. J. Physiol. Pathophysiol. Pharmacol..

