# Supplementary material for: An Integrated Multimodal-Based CAD System for Breast Cancer Diagnosis
Source: Cancers (Basel). 2024 Nov 5;16(22):3740. doi: 10.3390/cancers16223740 (PMC11591763; doi:10.3390/cancers16223740)
Supplement: Supplementary file 1 [file cancers-16-03740-s001.zip › cancers-3198661-supplementary.pdf]

Image Model:

Our model is designed for a multi-class classification task. Since our dataset is relatively small, and CNNs generally require a large amount of data to perform well, we utilized transfer learning with a VGG16 architecture pre-trained on ImageNet. We set the include_top parameter to False to leverage VGG16's powerful feature extraction capabilities while focusing on learning class-specific patterns in our dataset.

Additionally, we applied cross-validation to evaluate the model's performance across five folds of the data. In each fold, the dataset was split into training and validation sets, and the model was trained and validated iteratively. To assess the performance for each fold, we used multiple evaluation metrics, including accuracy, precision, recall, F1-score, and AUC-ROC.

The model was trained for 80 epochs in each fold using the Adam optimizer, sparse categorical cross-entropy loss function, and the SoftMax activation function to generate probabilities for the three output classes. First, we ran the model on the uncleaned image dataset. Second, we re-ran the model using the cleaned image dataset. Finally, we tested the infected side images for the best-performing case (cleaned or uncleaned). Detailed information regarding training performance for each fold and test results on unseen images is provided in the following tables.

1. **Model Trained with Unclean Images**

Table S1: Evaluation Metrics for Each Fold of Model Trained on Unclean Images Using Validation Data

Fold	accuracy	precision	recall	F1-score	AUC-ROC
1	0.5185	0.6646	0.5185	0.5247	0.7184
2	0.4630	0.4714	0.4630	0.4513	0.7026
3	0.5926	0.5869	0.5926	0.5583	0.7170
4	0.6111	0.6966	0.6111	0.6133	0.7515
5	0.5741	0.5964	0.5741	0.5783	0.7192

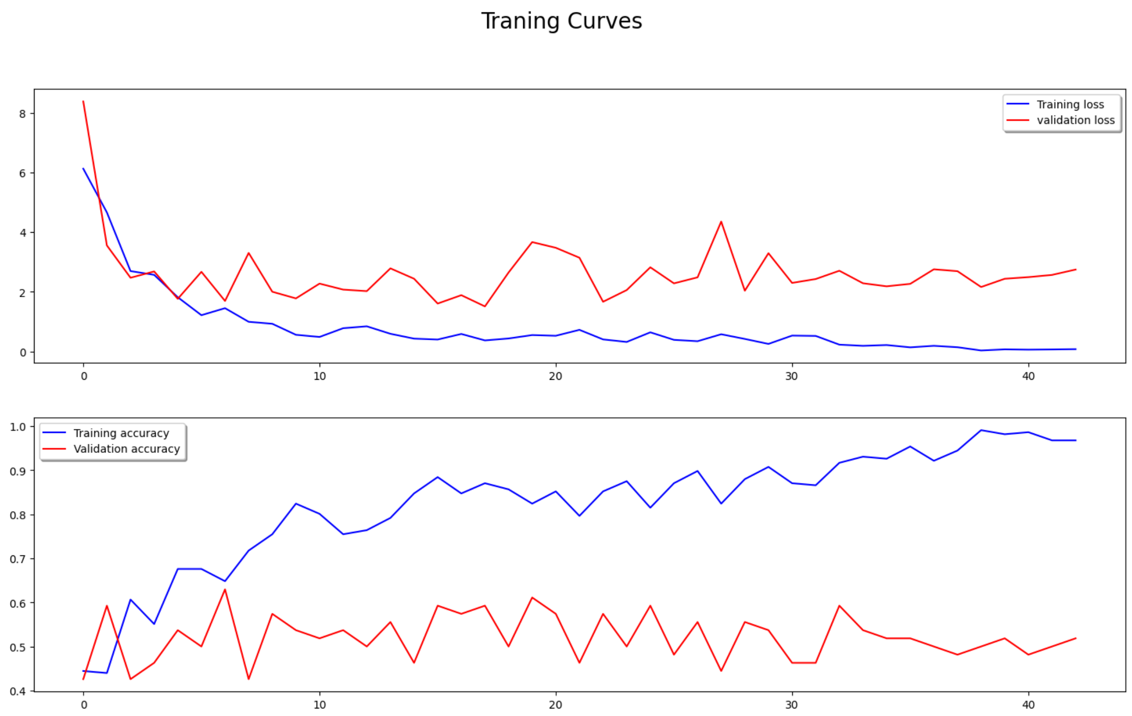

Figure S1: Training and Validation Curves for Uncleaned Images

Table S2: Results of Testing a Model Trained on Unclean Images Using Unseen Data

Accuracy	Precision	Recall	F1-score	AUC-ROC
0.500000	0.515873	0.500000	0.499167	0.714755

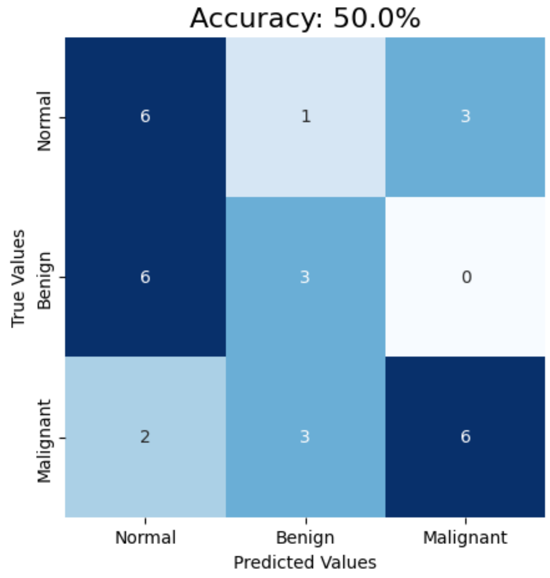

Figure S2: Confusion Matrix of a Model Trained on Unclean Images Tested with Unseen Data

Table S3: Predictions of a Model Trained on Unclean Images for Each Case in Unseen Testing Data

Sample	True Label	Predicted Label
1	Normal	Normal
2	Malignant	Malignant
3	Malignant	Benign
4	Malignant	Normal
5	Normal	Normal
6	Benign	Normal
7	Benign	Normal
8	Malignant	Malignant
9	Benign	Benign
10	Malignant	Malignant
11	Malignant	Benign
12	Benign	Normal
13	Normal	Normal
14	Normal	Malignant
15	Normal	Malignant
16	Malignant	Malignant
17	Normal	Normal
18	Malignant	Normal
19	Malignant	Malignant
20	Benign	Normal
21	Benign	Normal
22	Malignant	Benign
23	Normal	Benign
24	Benign	Benign
25	Normal	Normal
26	Normal	Malignant
27	Benign	Benign
28	Malignant	Malignant
29	Normal	Normal
30	Benign	Normal

2. Model Trained with clean Images

Table S4: Evaluation Metrics for Each Fold of Model Trained on Clean Images Using Validation Data

Fold	accuracy	precision	recall	F1-score	AUC-ROC
1	0.5370	0.5685	0.5370	0.5456	0.6999
2	0.6296	0.6987	0.6296	0.6216	0.7076
3	0.5926	0.6329	0.5926	0.5856	0.7217
4	0.5926	0.6714	0.5926	0.5956	0.7860
5	0.5926	0.6698	0.5926	0.5645	0.7204

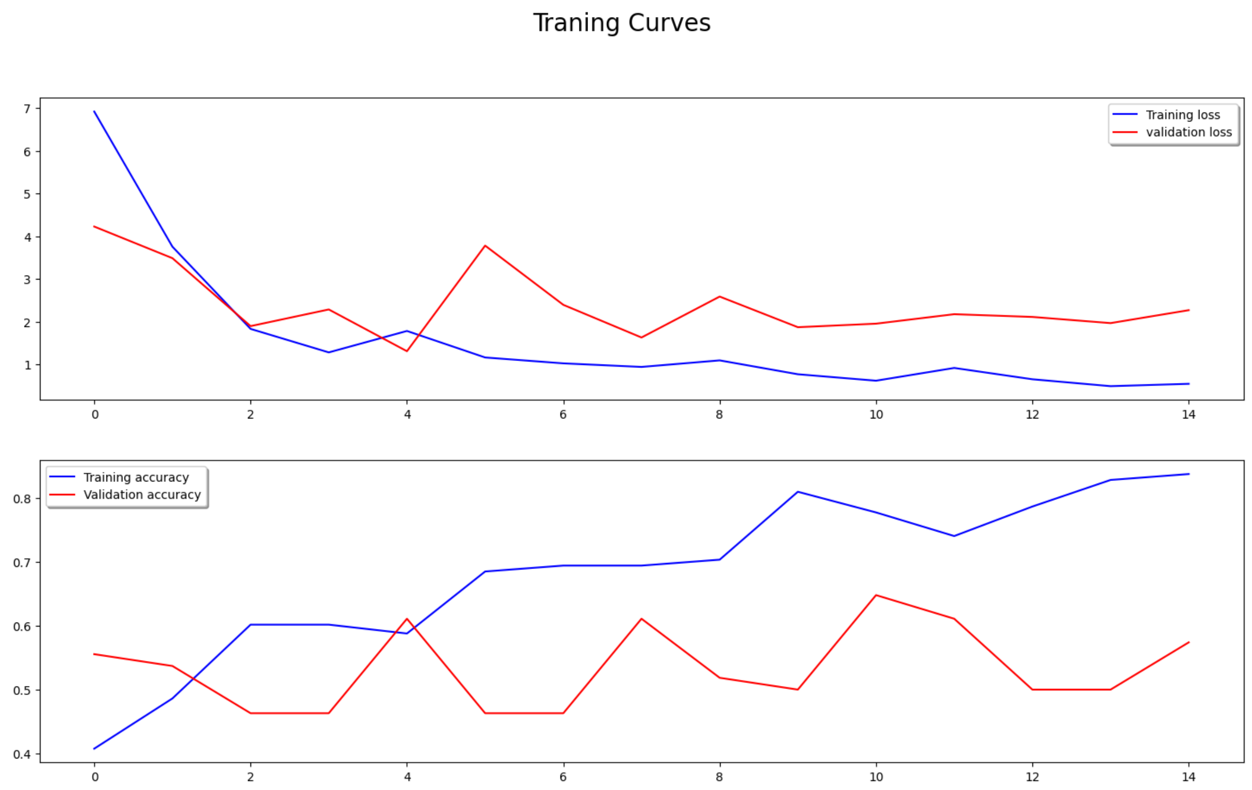

Figure S3: Training and Validation Curves for Cleaned Images

Table S5: Results of Testing a Model Trained on Clean Images Using Unseen Data

Accuracy	Precision	Recall	F1-score	AUC-ROC
0.566667	0.647619	0.566667	0.555556	0.713182

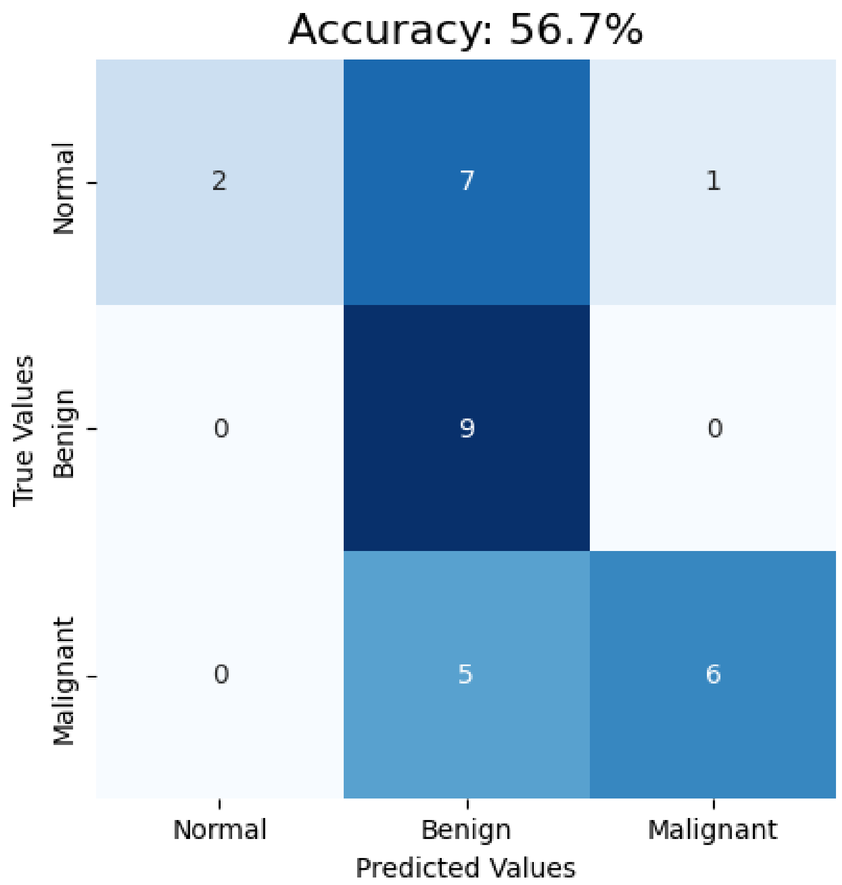

Figure S4: Confusion Matrix of a Model Trained on Clean Images Tested with Unseen Data

Table S6: Predictions of a Model Trained on Clean Images for Each Case in Unseen Testing Data

Sample	True Label	Predicted Label
1	Normal	Benign
2	Malignant	Malignant
3	Malignant	Benign
4	Malignant	Normal
5	Normal	Benign
6	Benign	Normal
7	Benign	Benign
8	Malignant	Malignant
9	Benign	Benign
10	Malignant	Malignant
11	Malignant	Benign
12	Benign	Benign
13	Normal	Normal
14	Normal	Benign
15	Normal	Benign
16	Malignant	Malignant
17	Normal	Normal

18	Malignant	Normal
19	Malignant	Malignant
20	Benign	Benign
21	Benign	Benign
22	Malignant	Benign
23	Normal	Benign
24	Benign	Benign
25	Normal	Normal
26	Normal	Malignant
27	Benign	Benign
28	Malignant	Malignant
29	Normal	Benign
30	Benign	Benign

3. Model Trained with Images of Infected Side Only

Table S7: Evaluation Metrics for Each Fold of Model Trained on Infected Side Images Using Validation Data

Fold	accuracy	precision	recall	F1-score	AUC-ROC
1	0.3704	0.4352	0.3704	0.3600	0.5815
2	0.5370	0.5399	0.5370	0.5161	0.6949
3	0.6296	0.6292	0.6296	0.6271	0.7822
4	0.5370	0.5567	0.5370	0.5309	0.7031
5	0.5556	0.6395	0.5556	0.5584	0.7206

Traning Curves

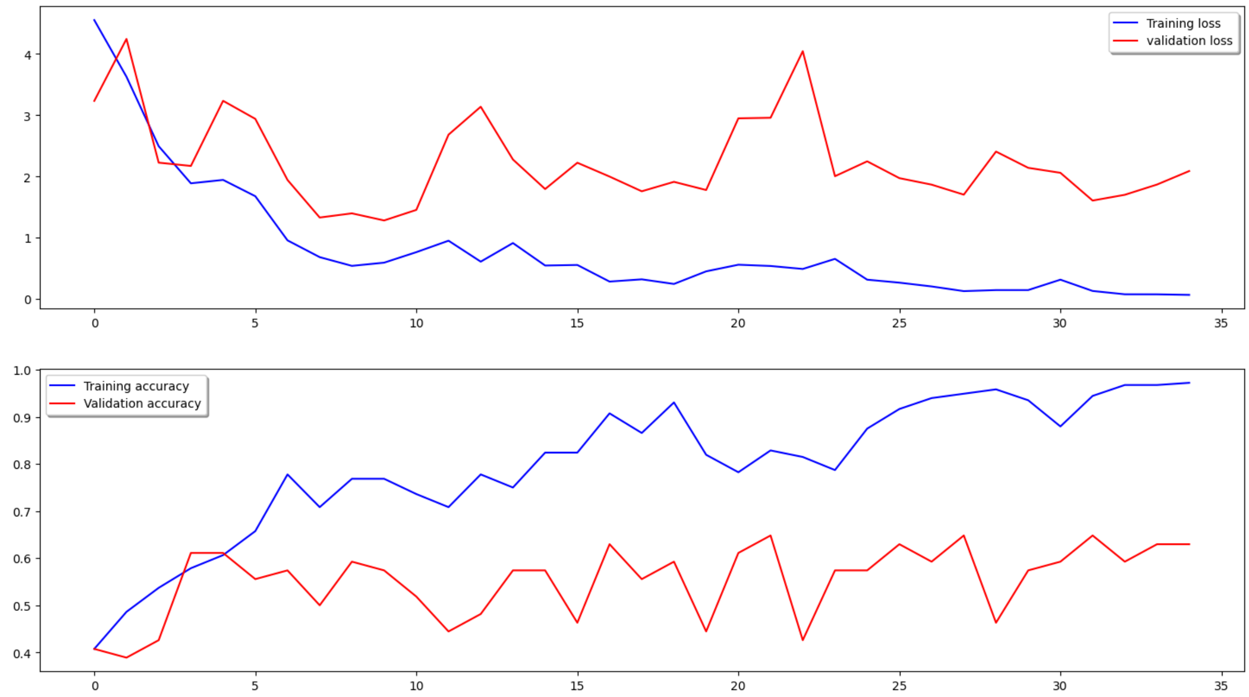

Figure S5: Training and Validation Curves for Infected Side Images

Table S8: Results of Testing a Model Trained on Infected Side Images Using Unseen Data

Accuracy	Precision	Recall	F1-score	AUC-ROC
0.433333	0.510498	0.433333	0.457143	0.680980

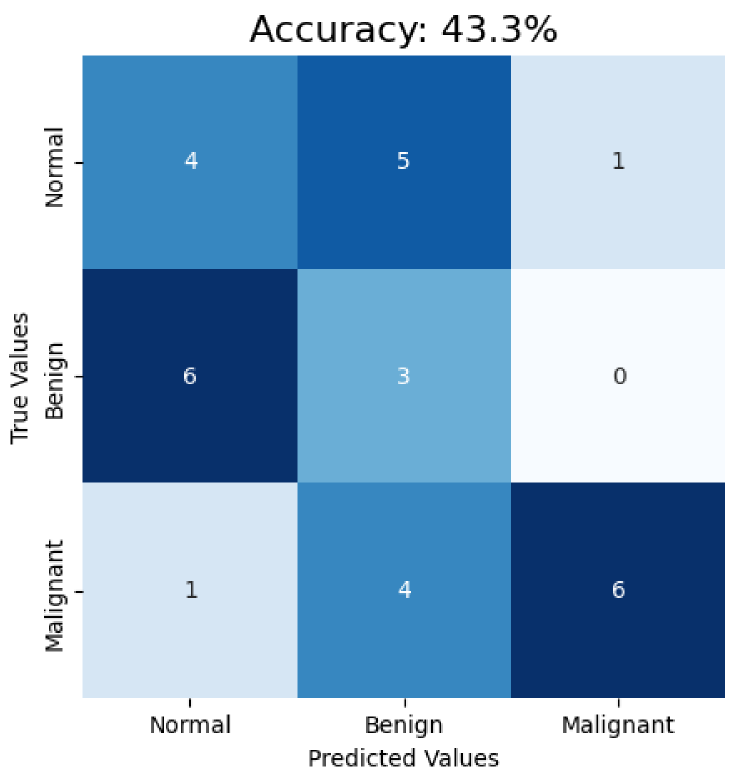

Figure S6: Confusion Matrix of a Model Trained on Infected Side Images Tested with Unseen Data

Table S9: Predictions of a Model Trained on Infected Side Images for Each Case in Unseen Testing Data

Sample	True Label	Predicted Label
1	Normal	Benign
2	Malignant	Malignant
3	Malignant	Benign
4	Malignant	Normal
5	Normal	Benign
6	Benign	Benign
7	Benign	Normal
8	Malignant	Malignant
9	Benign	Benign
10	Malignant	Malignant
11	Malignant	Benign
12	Benign	Normal
13	Normal	Normal
14	Normal	Benign
15	Normal	Normal
16	Malignant	Malignant
17	Normal	Normal
18	Malignant	Benign

19	Malignant	Malignant
20	Benign	Normal
21	Benign	Normal
22	Malignant	Benign
23	Normal	Benign
24	Benign	Normal
25	Normal	Normal
26	Normal	Malignant
27	Benign	Benign
28	Malignant	Malignant
29	Normal	Benign
30	Benign	Normal

4. A Comparison of Prediction Performance on Unseen Images Across Three Image Models

Table S10: Testing Results of Three Image Models (Clean Images, Unclean Images, and Infected-Side Only from Clean Images) on Unseen Data

Metrics	Unclean Image	Clean Image	Infected Side Only- Clean Image
accuracy	0.500000	0.500000	0.433333
precision	0.515873	0.562266	0.510498
recall	0.500000	0.500000	0.433333
F1-score	0.499167	0.515960	0.457143
AUC-ROC	0.714755	0.697452	0.680980

Table S11: Predictions of Three Image Models (Clean Images, Unclean Images, and Infected-Side Only from Clean Images) for Each Case in Unseen Test Data

Sample	True Label	Unclean Image	Clean Image	Infected Side Only- Clean Image
1	Normal	Normal	Benign	Benign
2	Malignant	Malignant	Malignant	Malignant
3	Malignant	Benign	Benign	Benign
4	Malignant	Normal	Normal	Normal
5	Normal	Normal	Benign	Benign
6	Benign	Normal	Normal	Benign
7	Benign	Normal	Benign	Normal
8	Malignant	Malignant	Malignant	Malignant
9	Benign	Benign	Benign	Benign
10	Malignant	Malignant	Malignant	Malignant
11	Malignant	Benign	Benign	Benign
12	Benign	Normal	Benign	Normal

13	Normal	Normal	Normal	Normal
14	Normal	Malignant	Benign	Benign
15	Normal	Malignant	Benign	Normal
16	Malignant	Malignant	Malignant	Malignant
17	Normal	Normal	Normal	Normal
18	Malignant	Normal	Normal	Benign
19	Malignant	Malignant	Malignant	Malignant
20	Benign	Normal	Benign	Normal
21	Benign	Normal	Benign	Normal
22	Malignant	Benign	Benign	Benign
23	Normal	Benign	Benign	Benign
24	Benign	Benign	Benign	Normal
25	Normal	Normal	Normal	Normal
26	Normal	Malignant	Malignant	Malignant
27	Benign	Benign	Benign	Benign
28	Malignant	Malignant	Malignant	Malignant
29	Normal	Normal	Benign	Benign
30	Benign	Normal	Benign	Normal

Statistical Information Model:

We built a simple feedforward neural network, specifically a multilayer perceptron (MLP), designed for our multiclass classification task. The input layer contains a number of neurons equal to the number of input features, while the output layer consists of three neurons, one for each class, utilizing the SoftMax activation function. The model includes two hidden layers with 16 and 32 neurons, respectively, both leveraging the ReLU activation function. For optimization, we used the Adam optimizer, and for the loss function, we applied sparse categorical cross-entropy.

Additionally, we employed five-fold cross-validation. To evaluate the model's performance during cross-validation, we used accuracy, precision, recall, F1-score, and AUC-ROC metrics to assess the model in each fold to get a comprehensive evaluation of its overall performance.

Furthermore, we trained traditional machine learning algorithms on statistical features, including decision tree (DT), random forest (RF), k-nearest neighbors (KNN), Naive Bayes (NB), and gradient boosting (GB). The following tables and figures illustrate and compare the performance of these models on the classification task using statistical data.

Table S12: Evaluation Metrics for Each Fold of the MLP Trained on Statistical Features with Validation Data

Fold	accuracy	precision	recall	F1-score	AUC-ROC
1	0.9074	0.9252	0.9074	0.9057	0.9553
2	0.9259	0.9276	0.9259	0.9246	0.9592
3	0.9815	0.9826	0.9815	0.9815	0.9943
4	0.9259	0.9297	0.9259	0.9260	0.9770
5	0.8519	0.9012	0.8519	0.8420	0.9183

Traning Curves

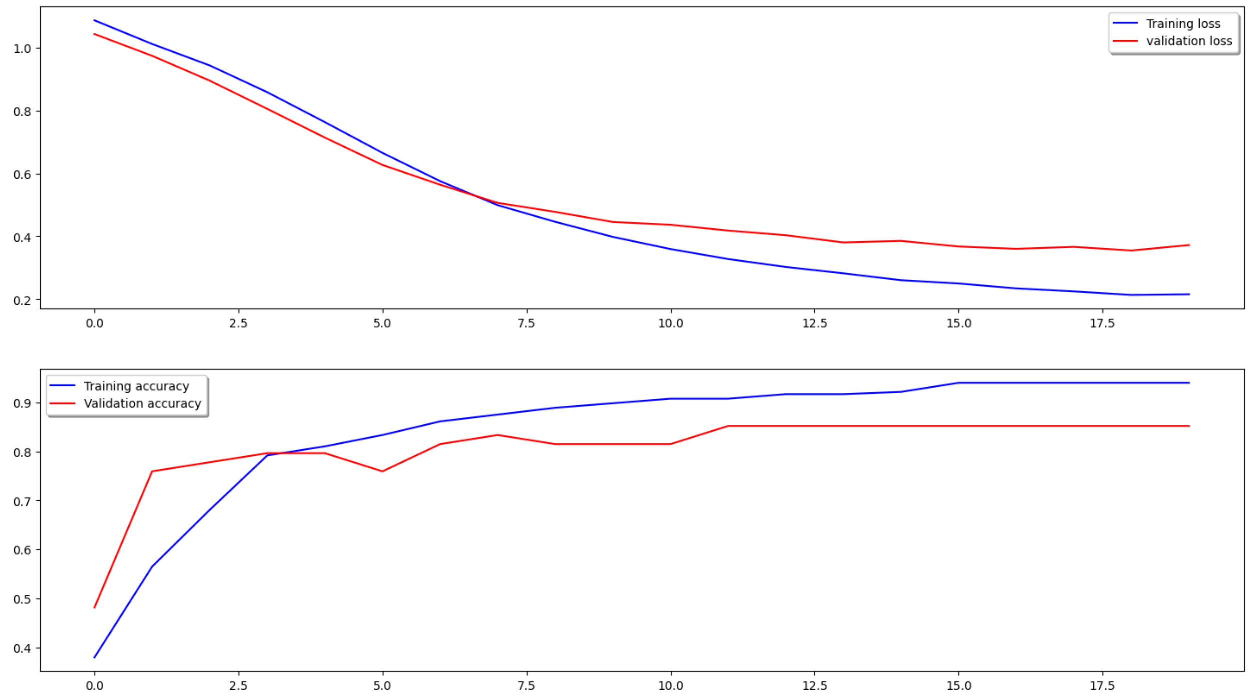

Figure S7: Training and Validation Curves for MLP

Table S13: Results of Testing DT, RF, NB, GB, KNN, and MLP Trained on Statistical Features Using Unseen Data

Metrics	DT	RF	KNN	NB	GB	MLP
accuracy	1.00	1.00	0.87	0.87	0.93	0.93
precision	1.00	1.00	0.88	0.87	0.95	0.94
recall	1.00	1.00	0.88	0.87	0.93	0.93
F1-score	1.00	1.00	0.87	0.86	0.94	0.93
AUC-ROC	1.00	1.00	0.96	0.94	1.00	0.98

Table S14: Predictions of DT, RF, NB, GB, KNN, and MLP Trained on Statistical Features for Each Case in Unseen Test Data

Sample	True Label	DT	RF	KNN	NB	GB	MLP
1	Normal	Normal	Normal	Normal	Normal	Malignant	Normal
2	Malignant	Malignant	Malignant	Normal	Normal	Malignant	Normal
3	Malignant	Malignant	Malignant	Malignant	Malignant	Malignant	Malignant
4	Malignant	Malignant	Malignant	Malignant	Malignant	Malignant	Malignant
5	Normal	Normal	Normal	Normal	Normal	Normal	Normal
6	Benign	Benign	Benign	Benign	Benign	Benign	Benign
7	Benign	Benign	Benign	Benign	Benign	Benign	Benign
8	Malignant	Malignant	Malignant	Benign	Malignant	Malignant	Malignant
9	Benign	Benign	Benign	Benign	Benign	Benign	Benign

10	Malignant	Malignant	Malignant	Malignant	Malignant	Malignant	Malignant
11	Malignant	Malignant	Malignant	Malignant	Benign	Malignant	Malignant
12	Benign	Benign	Benign	Benign	Benign	Benign	Benign
13	Normal	Normal	Normal	Normal	Normal	Normal	Normal
14	Normal	Normal	Normal	Normal	Normal	Normal	Normal
15	Normal	Normal	Normal	Benign	Normal	Normal	Normal
16	Malignant	Malignant	Malignant	Malignant	Benign	Malignant	Malignant
17	Normal	Normal	Normal	Normal	Normal	Normal	Normal
18	Malignant	Malignant	Malignant	Malignant	Malignant	Malignant	Malignant
19	Malignant	Malignant	Malignant	Malignant	Malignant	Malignant	Malignant
20	Benign	Benign	Benign	Benign	Benign	Benign	Benign
21	Benign	Benign	Benign	Benign	Benign	Benign	Benign
22	Malignant	Malignant	Malignant	Normal	Malignant	Malignant	Normal
23	Normal	Normal	Normal	Normal	Normal	Normal	Normal
24	Benign	Benign	Benign	Benign	Benign	Benign	Benign
25	Normal	Normal	Normal	Normal	Normal	Normal	Normal
26	Normal	Normal	Normal	Normal	Normal	Normal	Normal
27	Benign	Benign	Benign	Benign	Benign	Benign	Benign
28	Malignant	Malignant	Malignant	Malignant	Malignant	Malignant	Malignant
29	Normal	Normal	Normal	Normal	Normal	Malignant	Normal
30	Benign	Benign	Benign	Benign	Malignant	Benign	Benign

Combined Models:

1. **Soft Voting**

S In the soft voting technique, predictions are made based on a weighted average of multiple models. To ensure that both image-based and statistical features contribute fairly to the prediction, we initially divided the total weight evenly between these two feature types (50:50). Additionally, since both DT and RF perfectly predicted the test cases for the statistical dataset with no misclassifications, we decided to eliminate RF by assigning it a weight of zero.

In the first experiment, we distributed the 50% allocated to statistical features among the DT, KNN, NB, GB, and MLP classifiers, while assigning the remaining 50% to the CNN for image-based features. However, the performance was lower than expected due to the weaker performance of the CNN model. To address this, we reallocated 5% of the weight from CNN to the statistical classifiers, resulting in a new weight distribution of 55% for statistical classifiers and 45% for CNN. We aimed to maintain a balance between the image and statistical features, hence limiting the weight adjustment to 5-10%.

In the second experiment, we evenly distributed the 55% weight among the statistical classifiers, which improved the performance of the soft voting model. For further enhancement, in the third experiment, we assigned the additional 5% weight to a single statistical classifier at a time, instead of distributing it equally. This further improved the performance.

In the fourth experiment, we reduced the CNN's weight to 40% and allocated most of the remaining 60% among the three best-performing classifiers: DT, GB, and MLP, while assigning a smaller portion to KNN and NB. This led to improved results. Since DT, GB, and MLP performed almost equally, we fine-tuned the weight distribution among them in the fifth experiment, achieving the best overall performance.

The detailed weight distributions for each experiment are presented in TableS15, while the corresponding performance metrics are provided in TableS16. As shown in the tables, increasing the weight assigned to statistical features improved performance, but we ensured fairness by maintaining the involvement of both image-based and statistical features. Additionally, the success of soft voting depends heavily on the quality of the models included in the ensemble.

TableS17 illustrates how the soft voting technique enhances the performance of both statistical and image-based models. For example, case (2) was misclassified by MLP, NB, and KNN, but was correctly classified by soft voting. Moreover, most of the cases misclassified by the CNN model were correctly classified with soft voting.

Table S15: Weight Distribution Among Classifiers in Each Soft Voting Experiment

Experiment	Statistical Features						Images features
	DT	RF	KNN	NB	GB	MLP	CNN
1	0.1	0	0.1	0.1	0.1	0.1	0.5
2	0.105	0	0.105	0.105	0.105	0.105	0.475
3	0.1	0.1	0.1	0.1	0.1	0.125	0.475
4	0.1	0	0.1	0.1	0.15	0.15	0.4
5	0.15	0	0.1	0.05	0.15	0.131	0.4

Table S16: Prediction Performance for Each Soft Voting Experiment on Test Datasets

Metrics	Experiment(1)	Experiment(2)	Experiment(3)	Experiment(4)	**Experiment(5)**
accuracy	0.40	0.74	0.63	0.87	**0.90**
precision	0.46	0.80	0.83	0.91	**0.93**
recall	0.37	0.44	0.63	0.87	**0.90**
F1-score	0.37	0.37	0.57	0.87	**0.90**
AUC-ROC	0.97	0.97	0.98	1	**1**

Table S17: Comparison of Soft Voting Predictions with Individual Ensemble Models for Each Case in the Unseen Test Data

Sample	True Label	DT	GB	KNN	NB	MLP	CNN	Soft Voting
1	Normal	Normal	Malignant	Normal	Normal	Normal	Normal	Malignant
2	Malignant	Malignant	Malignant	Normal	Normal	Normal	Malignant	Malignant
3	Malignant	Malignant	Malignant	Malignant	Malignant	Malignant	Benign	Malignant
4	Malignant	Malignant	Malignant	Malignant	Malignant	Malignant	Normal	Malignant

5	Normal	Normal	Normal	Normal	Normal	Normal	Benign	Normal
6	Benign	Benign	Benign	Benign	Benign	Benign	Normal	Benign
7	Benign	Benign	Benign	Benign	Benign	Benign	Normal	Benign
8	Malignant	Malignant	Malignant	Benign	Malignant	Malignant	Malignant	Malignant
9	Benign	Benign	Benign	Benign	Benign	Benign	Benign	Benign
10	Malignant	Malignant	Malignant	Malignant	Malignant	Malignant	Malignant	Malignant
11	Malignant	Malignant	Malignant	Malignant	Benign	Malignant	Benign	Malignant
12	Benign	Benign	Benign	Benign	Benign	Benign	Normal	Benign
13	Normal	Normal	Normal	Normal	Normal	Normal	Normal	Normal
14	Normal	Normal	Normal	Normal	Normal	Normal	Benign	Normal
15	Normal	Normal	Normal	Benign	Normal	Normal	Normal	Normal
16	Malignant	Malignant	Malignant	Malignant	Benign	Malignant	Malignant	Malignant
17	Normal	Normal	Normal	Normal	Normal	Normal	Normal	Normal
18	Malignant	Malignant	Malignant	Malignant	Malignant	Malignant	Normal	Malignant
19	Malignant	Malignant	Malignant	Malignant	Malignant	Malignant	Malignant	Malignant
20	Benign	Benign	Benign	Benign	Benign	Benign	Normal	Benign
21	Benign	Benign	Benign	Benign	Benign	Benign	Benign	Benign
22	Malignant	Malignant	Malignant	Normal	Malignant	Normal	Benign	Malignant
23	Normal	Normal	Normal	Normal	Normal	Normal	Benign	Normal
24	Benign	Benign	Benign	Benign	Benign	Benign	Benign	Benign
25	Normal	Normal	Normal	Normal	Normal	Normal	Normal	Normal
26	Normal	Normal	Normal	Normal	Normal	Normal	Malignant	Normal
27	Benign	Benign	Benign	Benign	Benign	Benign	Benign	Benign
28	Malignant	Malignant	Malignant	Malignant	Malignant	Malignant	Malignant	Malignant
29	Normal	Normal	Malignant	Normal	Normal	Normal	Benign	Normal
30	Benign	Benign	Benign	Benign	Malignant	Benign	Normal	Benign

2. Hard Voting

The hard voting method outputs the most common class predicted among the ensemble models. Since we have only one model trained on images and six models trained on statistical features, we mitigated potential bias by including the lower-performing statistical model, NB, alongside MLP and CNN. Table S18 shows the hard voting performance, and Table S19 illustrates how hard voting improves the model and reduces misclassification, even though some correct predictions by the CNN were misclassified by hard voting.

Table S18: prediction performance of hard voting on test dataset

Accuracy	Precision	Recall	F1-score
0.93	0.94	0.94	0.94

Table S19: Comparison of Hard Voting Predictions with Individual Ensemble Models for Each Case in the Unseen Test Data

Sample	True Label	NB	MLP	CNN	Soft Voting
1	Normal	Normal	Normal	Normal	Normal

2	Malignant	Normal	Normal	Malignant	Normal
3	Malignant	Malignant	Malignant	Benign	Malignant
4	Malignant	Malignant	Malignant	Normal	Malignant
5	Normal	Normal	Normal	Benign	Normal
6	Benign	Benign	Benign	Normal	Benign
7	Benign	Benign	Benign	Normal	Benign
8	Malignant	Malignant	Malignant	Malignant	Malignant
9	Benign	Benign	Benign	Benign	Benign
10	Malignant	Malignant	Malignant	Malignant	Malignant
11	Malignant	Benign	Malignant	Benign	Malignant
12	Benign	Benign	Benign	Normal	Benign
13	Normal	Normal	Normal	Normal	Normal
14	Normal	Normal	Normal	Benign	Normal
15	Normal	Normal	Normal	Normal	Normal
16	Malignant	Benign	Malignant	Malignant	Malignant
17	Normal	Normal	Normal	Normal	Normal
18	Malignant	Malignant	Malignant	Normal	Malignant
19	Malignant	Malignant	Malignant	Malignant	Malignant
20	Benign	Benign	Benign	Normal	Benign
21	Benign	Benign	Benign	Benign	Benign
22	Malignant	Malignant	Normal	Benign	Malignant
23	Normal	Normal	Normal	Benign	Normal
24	Benign	Benign	Benign	Benign	Benign
25	Normal	Normal	Normal	Normal	Normal
26	Normal	Normal	Normal	Malignant	Normal
27	Benign	Benign	Benign	Benign	Benign
28	Malignant	Malignant	Malignant	Malignant	Malignant
29	Normal	Normal	Normal	Benign	Normal
30	Benign	Malignant	Benign	Normal	Malignant

3. Concatenation

We built a hybrid deep learning model that combines both image-based and statistical features. The model integrates a CNN model with a pre-trained VGG19 network and an MLP model for processing statistical data. The output from the VGG19 feature extractor is concatenated with the statistical features using a Concatenate layer, creating a merged feature representation from both types of inputs. This combined representation is then passed through a Softmax activation function for multi-class classification. The model is trained using the Adam optimizer and the sparse categorical cross-entropy loss function. Training is performed for up to 100 epochs, with early stopping if no improvement is seen for 10 consecutive epochs. Additionally, we applied 5-fold cross-validation and evaluated the accuracy, precision, recall, F1-score, and AUC-ROC on the validation data for each fold during the cross-validation.

The model performed optimally, and the learning process progressed smoothly. The loss curve for both training and validation accuracy converged within the first 5 epochs, and the accuracies of both reached convergence by the 30th epoch. Figure 8 displays the training and loss curves.

Additionally, Table S20 highlights the strong validation results for each epoch, with accuracies generally ranging between 0.78 and 0.95. Table S21 presents the results of testing the model on unseen data, which show even better performance than the validation phase. This indicates that the model is highly effective in correctly classifying the three classes in our dataset. For detailed results of each test case, see Table S22.

Table S20: Evaluation of concatenation on validation data for each fold

Fold	accuracy	precision	recall	F1-score	AUC-ROC
1	0.9074	0.9275	0.9074	0.9051	0.9509
2	0.8148	0.8130	0.8148	0.8123	0.9136
3	0.8889	0.8889	0.8889	0.8889	0.9635
4	0.7778	0.7778	0.7778	0.7778	0.8904
5	0.8519	0.8581	0.8519	0.8522	0.9434

Table S21:Results of Testing a Model Trained on Merged Image and Statistical Features Using Unseen Data

Accuracy	Precision	Recall	F1-score	AUC-ROC
0.9333	0.9364	0.9333	0.9331	0.9791

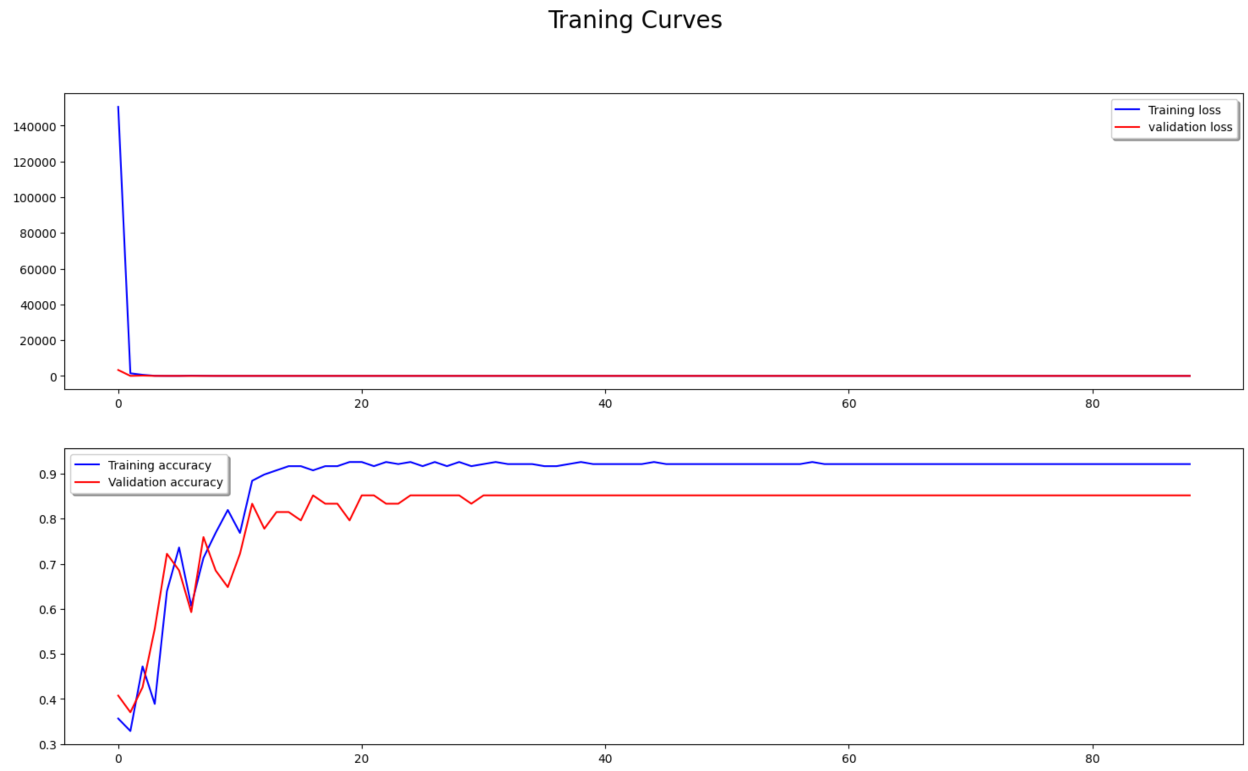

Figure S8: Training and Validation Curves for Concatenated Feature Model

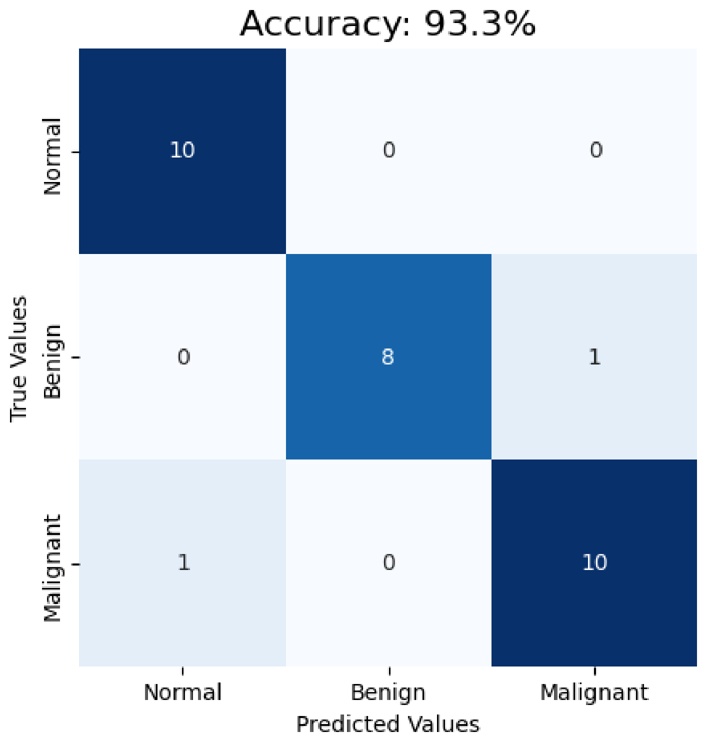

Figure S9: Confusion Matrix of a Model Trained on Concatenated Features Tested with Unseen Data

Table S22: Comparison of Predictions from a Model Trained on Concatenated Image and Statistical Features with Models Trained Separately on Image (CNN) and Statistical Features (MLP) for Each Case in the Unseen Test Data

Sample	True Label	MLP	CNN	Concatenation
1	Normal	Normal	Normal	Normal
2	Malignant	Normal	Malignant	Normal
3	Malignant	Malignant	Benign	Malignant
4	Malignant	Malignant	Normal	Malignant
5	Normal	Normal	Benign	Normal
6	Benign	Benign	Normal	Benign
7	Benign	Benign	Normal	Benign
8	Malignant	Malignant	Malignant	Malignant
9	Benign	Benign	Benign	Benign
10	Malignant	Malignant	Malignant	Malignant
11	Malignant	Malignant	Benign	Malignant
12	Benign	Benign	Normal	Benign
13	Normal	Normal	Normal	Normal
14	Normal	Normal	Benign	Normal
15	Normal	Normal	Normal	Normal
16	Malignant	Malignant	Malignant	Malignant
17	Normal	Normal	Normal	Normal
18	Malignant	Malignant	Normal	Malignant

19	Malignant	Malignant	Malignant	Malignant
20	Benign	Benign	Normal	Benign
21	Benign	Benign	Benign	Benign
22	Malignant	Normal	Benign	Malignant
23	Normal	Normal	Benign	Normal
24	Benign	Benign	Benign	Benign
25	Normal	Normal	Normal	Normal
26	Normal	Normal	Malignant	Normal
27	Benign	Benign	Benign	Benign
28	Malignant	Malignant	Malignant	Malignant
29	Normal	Normal	Benign	Normal
30	Benign	Benign	Normal	Malignant

4. Comparison between Soft Voting, Hard Voting, and Concatenation Approaches

Our experiments prove that merging statistical features with image features boosts the performance of Computer-Aided Diagnosis (CAD) systems for breast cancer. All three experimental approaches for combining statistical and image features (hard voting, soft voting, and concatenation) show enhanced performance. Additionally, when analyzing the test cases, we noticed that they mostly corrected the same misclassified cases that were missed by the statistical-based or image-based models individually. This enhancement primarily strengthens the image features, but our experiments show that it also improves the contribution of the statistical features. The diagnosis and detection of breast cancer are heavily influenced not only by mammogram images but also by demographic and historical health data.

Table S23: Comparison Between the Combined Features Approaches: Hard Voting, Soft Voting, and Concatenation on the Test Data

Metrics	**Soft Voting**	Hard Voting	Concatenation
accuracy	0.90	0.93	0.93
precision	0.93	0.94	0.94
recall	0.90	0.94	0.93
F1-score	0.90	0.94	0.93

Validation of Our Approach:

In our study, we aim to validate our novel approach that combines statistical data with mammogram images to enhance computer-aided diagnosis systems. Given that our data is novel and has not been previously reported in the literature, we cannot validate our approach against benchmark datasets or compare it with established methods for breast cancer diagnosis.

To assess the effectiveness of our model, we applied cross-validation across experiments, ensuring robust performance evaluation. This approach allows us to be confident that the performance

results are not biased or influenced by data selection, as each model is trained and validated on different subsets of the data. Additionally, our team includes a physician who collaborates with us on the data, providing valuable insights and expertise that further enhance the precision of our analysis.

This process qualifies as an ablation study because we conducted six experiments designed to systematically evaluate the contributions of different components to the overall performance of our approach. Specifically, these experiments allowed us to assess:

- The effectiveness of using statistical data alone in diagnosis.
- The impact of utilizing unclean images, which contain noise and additional information such as doctor's notes.
- The performance of clean images, which are free of extraneous information.
- The benefits of combining models through different approaches, including soft voting, hard voting, and feature concatenation, to enhance diagnostic accuracy.

By analyzing these distinct scenarios, we gained insights into how each component influences the model’s predictive capabilities.

Our findings indicate that the model trained on clean images outperformed the one trained on unclean images. Additionally, integrating images and statistical information enhanced performance compared to using images alone. We employed different approaches for combining the models, and all combinations outperformed the individual use of images or statistical data, particularly in certain test cases related to the statistical models. This further demonstrates that combining the data is effective in improving diagnostic accuracy.

Confidence Intervals

To further validate our results, we calculated 95% confidence intervals for each performance metric (accuracy, precision, recall, F1-score, and AUC-ROC) using 5-fold cross-validation (see Figure 10). This method estimates performance metrics across different subsets of the training data that includes concatenated features, providing a reliable assessment by averaging the results from each fold.

The confidence intervals indicate the uncertainty around these metrics, showing the range within which the true performance of the model is likely to fall. For example, an accuracy metric with a 95% confidence interval of 80.17% to 89.46% suggests that the model's true accuracy lies within this range when applied to similar datasets.

The narrowness of the confidence intervals indicates consistent performance across various scenarios, which is crucial in the medical field where reliable diagnoses can significantly impact patient outcomes. Overall, the 95% confidence intervals substantiate the robustness of our findings, underscoring the model's effectiveness in predicting breast cancer outcomes. This validation enhances the credibility of our approach and highlights its applicability in clinical settings, ultimately contributing to improved patient care.

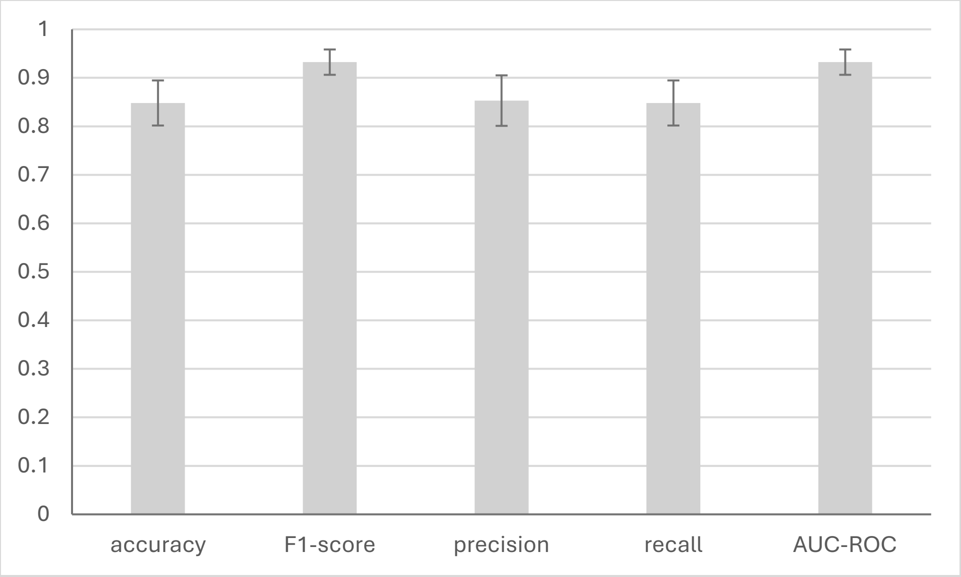

Figure S10: Performance Metrics Overview: Error Bars Representing 95% Confidence Intervals

5-Folds Cross-Validation Paired t-Test

Due to the various sources of random variation in the training, testing, and validation data, we applied a paired t-test on the accuracy obtained from 5-fold cross-validation to verify the significance of differences between two models. This approach strengthens the validation of our work by ensuring that the differences observed in our experiments are meaningful. We performed pairwise combinations as mentioned in Table S1, focusing on the main models that support our findings.

Table S24: Combination for Pairwise t-Test

Model	Statistical MLP	One-side image CNN	Unclean Image CNN	Clean Image CNN	Concatenated features
Statistical MLP					
Unclean Image CNN				✓	
Clean Image CNN		✓			✓
One-side image CNN					
Concatenated features					

– *Model Trained with Clean Images vs. Model Trained with Unclean Images:*

The p-value is 0.326186, indicating no significant difference between these models. However, our experiments demonstrate a positive difference in performance metrics based on testing results: accuracy improved from 50% to 56.7%, and the F1-Score increased from 49.4% to 55.5%. This

indicates that, while the p-value suggests statistical insignificance, there is a notable enhancement in model performance on the testing dataset.

– *Model Trained with Clean Images vs. Model Trained with One-Sided Images:*

The p-value is 0.132694, suggesting no statistical difference. Nonetheless, our experiments show a drop in prediction accuracy for one-sided images to 43.3% on the testing dataset. While the difference between models trained on different image types may not be statistically significant, it significantly affects performance on unseen data.

– *Model Trained with Clean Images vs. Model Trained with Concatenated Features:*

This comparison reveals a significant difference with a p-value of 0.001798, indicating that the improvement from integrating image features with statistical features is substantial. This finding reinforces the validity of our approach.

Overall, while some model comparisons show no statistically significant differences, the practical implications of these differences on performance metrics highlight the importance of the chosen data types. The significant improvement from integrating features further validates our methodology in enhancing diagnostic accuracy.
